# Extracellular RNA in a single droplet of human serum reflects physiologic and disease states

**DOI:** 10.1073/pnas.1908252116

**Published:** 2019-09-03

**Authors:** Zixu Zhou, Qiuyang Wu, Zhangming Yan, Haizi Zheng, Chien-Ju Chen, Yuan Liu, Zhijie Qi, Riccardo Calandrelli, Zhen Chen, Shu Chien, H. Irene Su, Sheng Zhong

**Affiliations:** ^a^Department of Bioengineering, University of California San Diego, La Jolla, CA 92093;; ^b^Genemo Inc., San Diego, CA 92121;; ^c^Institute of Engineering in Medicine, University of California San Diego, La Jolla, CA 92093;; ^d^Department of Diabetes Complications and Metabolism, Beckman Research Institute, Duarte, CA 91010;; ^e^Moores Cancer Center, University of California San Diego, La Jolla, CA 92093;; ^f^Department of Obstetrics, Gynecology and Reproductive Sciences, University of California San Diego, La Jolla, CA 92093

**Keywords:** extracellular RNA, biomarker, age, breast cancer, cancer recurrence

## Abstract

Extracellular RNAs (exRNAs) are present in human serum. It remains unclear to what extent these circulating exRNAs may reflect human physiologic and disease states. Here, we developed SILVER-seq (Small Input Liquid Volume Extracellular RNA Sequencing) to efficiently sequence both integral and fragmented exRNAs from a small droplet (5 μL to 7 μL) of liquid biopsy. We calibrated SILVER-seq in reference to other RNA sequencing methods based on milliliters of input serum and quantified droplet-to-droplet and donor-to-donor variations. We carried out SILVER-seq on more than 150 serum droplets from male and female donors ranging from 18 y to 48 y of age. SILVER-seq detected exRNAs from more than a quarter of the human genes, including small RNAs and fragments of mRNAs and long noncoding RNAs (lncRNAs). The detected exRNAs included those derived from genes with tissue (e.g., brain)-specific expression. The exRNA expression levels separated the male and female samples and were correlated with chronological age. Noncancer and breast cancer donors exhibited pronounced differences, whereas donors with or without cancer recurrence exhibited moderate differences in exRNA expression patterns. Even without using differentially expressed exRNAs as features, nearly all cancer and noncancer samples and a large portion of the recurrence and nonrecurrence samples could be correctly classified by exRNA expression values. These data suggest the potential of using exRNAs in a single droplet of serum for liquid biopsy-based diagnostics.

## Significance

The SILVER-seq technology enables sequencing extracellular RNAs (exRNAs) from a single droplet of liquid biopsy. This study revealed strong associations between serum exRNA expression levels and the donor’s sex and age. SILVER-seq detected serum exRNAs from the genes that are only expressed in brain, suggesting the possibility of monitoring brain gene expression from a blood test. Classifiers based on exRNA expression levels were able to separate breast cancer patients from control donors. The exRNA-based classifiers could also distinguish the patients with recurrent cancer from other breast cancer patients. The SILVER-seq technology can therefore lead the way to future in vitro diagnostics trials based on finger prick blood, which is more accessible for screening and frequent monitoring of human diseases.

Liquid biopsy is a rapidly expanding class of in vitro diagnostics (IVD) due to its accessibility (1). Nearly all types of molecular and cellular components in human blood have been explored as candidate targets for IVD development. These include circulating tumor cells, exosomes, extracellular proteins, peptides, hormones, metabolites, extracellular DNA and their methylated and hydroxymethylated forms, and extracellular RNAs (exRNAs) (2, 3).

A variety of exRNAs have been detected in human plasma and serum (4, 5). Small exRNAs including micro RNAs (miRNAs) have been correlated with clinical outcomes (6, 7). Less is known about the existence of other types of exRNAs and their relevance to clinical outcomes (4). To effectively analyze exRNA, we developed a low-input exRNA sequencing technology called Small Input Liquid Volume Extracellular RNA Sequencing (SILVER-seq). SILVER-seq takes as few as several microliters of serum as input. This volume is smaller than the typical yield of a finger prick, which is approximately 30 μL of blood. Based on the serum samples collected by the Predictors of Ovarian Insufficiency in Young Breast Cancer Patients study (8), we assessed the size distribution of serum exRNAs, carried out exRNA sequencing from over 130 serum samples, and assessed the correlations of different classes of serum exRNAs with physiological factors and clinical outcomes.

## Results

### Concentration and Size Distribution of exRNA in Human Serum.

We started by measuring the range of concentrations and sizes of exRNA in human serum. To this end, we analyzed 10 serum samples. To account for technical variability, we purified exRNA with 4 different RNA purification kits, including exoRNeasy, TRIzol LS, NORGEN, and QIAzol, and subsequently quantified them with a bioanalyzer. The measured exRNA concentrations ranged from 0.3 ng/mL to 4.2 ng/mL in these serum samples (*SI Appendix*, Fig. S1*A*). Most detected exRNA are within the size range of 20 nucleotides (nt) to 200 nt (*SI Appendix*, Fig. S1*B*). These data suggest that the exRNA concentrations are approximately several nanograms per milliliter and are either small RNAs or fragmented long RNAs in human serum.

### SILVER-seq for exRNA Sequencing.

We developed the SILVER-seq technique for exRNA sequencing, by adapting the major steps of single-cell RNA sequencing that also dealt with a small amount of input materials (9, 10). Unlike other liquid biopsy RNA sequencing (RNA-seq) methods, SILVER-seq does not start with RNA purification, because this would cause the loss of most RNA from the very small amount of serum. Instead, SILVER-seq involves adding library preparation reagents directly into the original liquid sample (*SI Appendix*, Fig. S2).

**Fig. 1.**
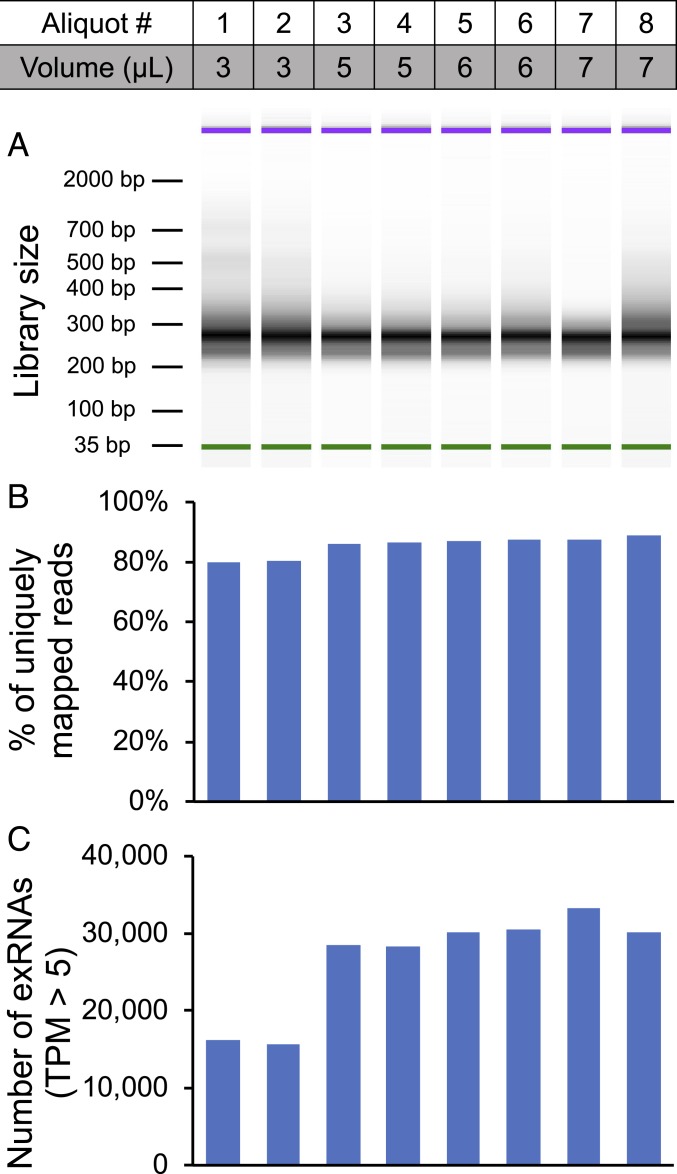
SILVER-seq sequencing libraries. (*A*) Size distribution of SILVER-seq constructed sequencing library from each serum aliquot (column), indexed by 1 to 8 (Aliquot #). Volume (microliters) is the volume of each aliquot. (*B*) Percentage of uniquely mapped reads of the corresponding library (column). (*C*) Number of exRNAs with 5 or more TPM in each library (column).

To test whether SILVER-seq could reliably produce sequencing libraries from microliters of human serum, we split a serum sample into 8 aliquots, with the volumes of 3, 5, 6, and 7 μL, respectively, in replicates. The final sequencing libraries ranged in fragment size from approximately 200 base pairs (bp) to 300 bp (Fig. 1*A*). This size range was consistent with the expectation, considering the 20- to 200-nt exRNA plus several nucleotides of template switching oligos and 2 sequencing adaptors totaling 132 bp. We sequenced the 8 libraries to yield an average of 4.8 million single-end sequencing reads per library. More than 80% of the reads from each library were uniquely mapped to the human genome (hg38) (Fig. 1*B*). These data suggest that SILVER-seq could consistently generate sequencing libraries from a few microliters of human serum.

### Sensitivity Analysis of Input Volumes.

To evaluate the impact of input volume on the quality of the sequencing library, we used the sequence mapping rate and the number of mapped exRNAs as 2 metrics to reflect the quality of a sequencing library. While 5 μL to 7 μL of input (aliquots 3 to 8) resulted in 80% or higher mapping rates and similar numbers of mapped exRNAs, 3 μL of input (aliquots 1 and 2) resulted in smaller mapping rates and fewer detected exRNAs (Fig. 1 *B* and *C*). To test the donor effect on library quality, we analyzed additional serum samples from 2 other donors (donors 2 and 3). We split the serum from donor 2 into four 3-μL and two 7-μL aliquots, and split the serum from donor 3 into five 3-μL and four 7-μL aliquots, resulting in a total of 15 serum aliquots. We constructed a SILVER-seq library from each serum aliquot and sequenced each library to yield approximately 5 million reads. The mapping rates from 7 μL-derived libraries were again higher than those from 3 μL-derived libraries (*SI Appendix*, Fig. S3*A*) with more detected exRNAs (*SI Appendix*, Fig. S3*B*). These data from the 2 additional donors reinforced the idea that SILVER-seq can produce sequencing libraries from microliters of input serum, and suggest 5 μL to 7 μL as the preferred input volume for SILVER-seq.

### Comparison of SILVER-seq and Standard RNA-seq.

We compared the exRNA expression profiles obtained using SILVER-seq with those obtained using standard RNA-seq methods. The expected amount of exRNA in 5 μL to 7 μL of serum (SILVER-seq input volume) is approximately 10 pg, comparable to the amount of RNA in a single cell (11). Given the poor correlation between gene expression quantified by single-cell and bulk RNA-seq (12), we did not anticipate a strong correlation between exRNA expression levels measured from several microliters of serum (SILVER-seq) and those from several milliliters (standard RNA-seq).

We examined the overlaps of detected exRNAs between 2 experiments. To establish the exRNAs that can be detected by 2 standard RNA-seq experiments, we purified and sequenced RNA from 2 serum samples from the same donor (RNA-seq-1 and RNA-seq-2), which detected 2,379 and 4,500 exRNAs, respectively, with 563 exRNAs in the intersection. Next, we applied SILVER-seq to 7 μL of serum from the same donor. SILVER-seq detected 20,841 exRNAs, of which 1,706 and 2,933 intersected with the exRNAs detected in RNA-seq-1 and RNA-seq-2, respectively (*SI Appendix*, Fig. S4 *A* and *B* and Table S1 *A* and *B*). A gene detected by either RNA-seq-1 or RNA-seq-2 has a 4.5-fold increase of odds to be detected by SILVER-seq (odds ratio = 4.5, χ^2^ *P* value < 10^−32^) (*SI Appendix*, Table S1*C*). Furthermore, a gene detected by both RNA-seq-1 and RNA-seq-2 has a 6.9-fold increase of odds to be detected by SILVER-seq (odds ratio = 6.9, χ^2^ *P* value < $10^{-32}$) (*SI Appendix*, Table S1*D*). Therefore, exRNAs detected by standard RNA-seq are more likely to be detected by SILVER-seq than those undetectable by the standard RNA-seq. Furthermore, the exRNAs detected by both standard RNA-seq assays are even more likely to be detected by SILVER-seq.

Next, we compared the measured exRNA expression levels. As a reference, Pearson correlation between the exRNA expression levels derived from RNA-seq-1 and RNA-seq-2 was 0.68 (*SI Appendix*, Fig. S4*C*). In comparison, the Pearson correlation was 0.67 between RNA-seq-1 and SILVER-seq, and 0.84 between RNA-seq-2 and SILVER-seq (*SI Appendix*, Fig. S4*C*). Thus, the correlation of the measured expression levels between SILVER-seq and a standard RNA-seq was comparable to the correlation between 2 standard RNA-seq methods.

### Variability of SILVER-seq Measurements among Biological Replicates.

We also assessed the variability of SILVER-seq measurements based on 2 serum aliquots of the same donor. Considering the stochasticity in splitting the pool of a small number of molecules (13), we anticipated large differences between 2 serum droplets.

We assayed two 7-μL serum aliquots with SILVER-seq and a 1-mL serum sample from the same donor by standard RNA-seq (*SI Appendix*, Fig. S4*D*). An exRNA detected by either SILVER-seq assay exhibited a 6.4- and 5.5-fold increased odds of being detected by standard RNA-seq (odds ratio = 6.4 and 5.5, χ^2^ *P* value < $10^{-32}$ for both cases) (*SI Appendix*, Table S2). An exRNA detected by both SILVER-seq assays exhibited a 6.2-fold increased odds for being detected by standard RNA-seq (odds ratio = 6.2, χ^2^ *P* value < $10^{-32}$). In this test, SILVER-seq–detected exRNAs are more likely to be detected by standard RNA-seq. However, adding replicate SILVER-seq assays did not further increase overlaps with standard RNA-seq, likely reflecting droplet-to-droplet biological variability.

Next, we compared the measured exRNA expression levels. The Pearson correlation was 0.66 between the 2 SILVER-seq assays, and 0.64 and 0.85 between SILVER-seq and each standard RNA-seq assay (*SI Appendix*, Fig. S4 *C* and *E*–*G*). In this test, the correlation between 2 SILVER-seq assays was comparable to the correlation between a SILVER-seq and a standard RNA-seq.

### An Estimate of Total Number of exRNAs in Serum.

We tested whether the number of detected exRNAs will increase as we combine SILVER-seq data of serum aliquots from the same donor. To this end, we analyzed 2 donors and prepared 15 serum aliquots from each donor. We carried out SILVER-seq from every aliquot. The SILVER-seq of the first aliquot of each donor was mapped to approximately 30,000 genes (*SI Appendix*, Fig. S5). As we sequentially combined SILVER-seq data of additional aliquots, these numbers increased and plateaued at ∼41,000 genes, which is 67.6% of the annotated coding and noncoding genes of the human genome (hg38). These data suggest that not all genes gave rise to exRNAs in serum. Each SILVER-seq based on 7 μL of serum could detect approximately 3/4 of the exRNAs that were detectable by pooling the SILVER-seq data from repeated assays (*SI Appendix*, Fig. S5).

### Presence of exRNAs Derived from Tissue-Specific Genes.

**Fig. 2.**
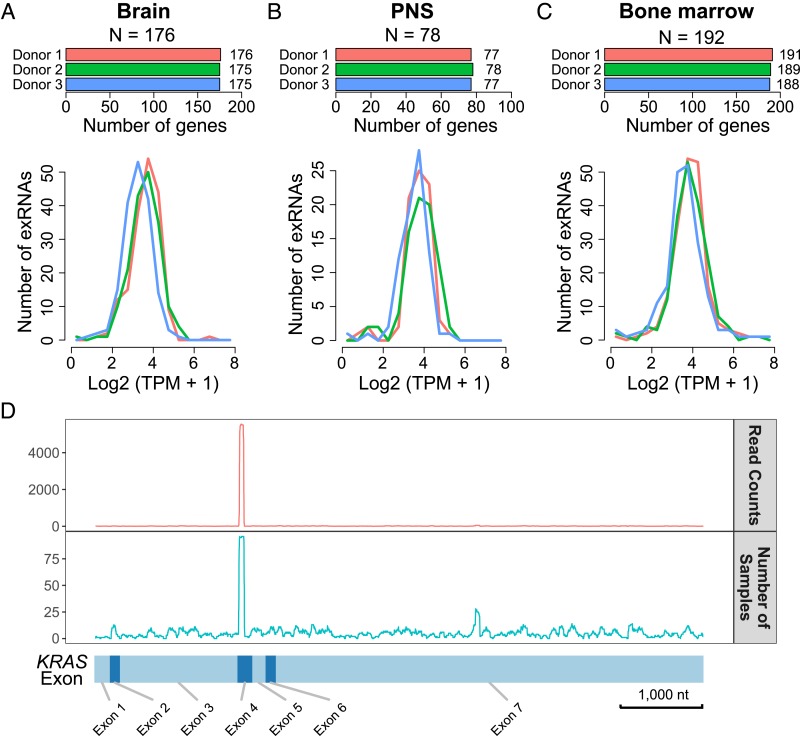
Presence of exRNAs derived from genes with tissue-specific expression. (*A*–*C*) Number and expression levels of the exRNAs derived from (*A*) brain-, (*B*) PNS-, and (*C*) bone marrow-specific genes. (*Upper*) The number of detected exRNAs in each donor. N, the total number of genes that are specifically expressed in this tissue. (*Lower*) Distribution of the expression of the exRNAs derived from the corresponding tissue-specific genes. (*D*) Distribution of SILVER-seq reads on all of the KRAS exons (*x* axis). (*Upper*) Cumulative read counts from all serum samples. (*Lower*) The number of serum samples with reads mapped to respective KRAS exons.

We tested whether tissue-specific gene expression contributed to exRNA in circulation. To this end, we used previously reported genes with tissue-specific expressions, including 176, 78, and 192 genes that are specifically expressed in brain, peripheral nervous system (PNS), and bone marrow, respectively (14, 15). With the exception of 1 brain-specific and 3 bone marrow-specific genes, exRNAs derived from all of the tissue-specific genes were detected in all 3 donors (Fig. 2 *A*–*C*, *Upper*). Furthermore, the expression levels as measured by transcripts per million (TPM) were not concentrated near 0 (Fig. 2 *A*–*C*, *Lower*). Instead, the exRNA abundances (TPM) of tissue-specific genes exhibited unimodal distributions with positive modes (*P* value < $10^{-32}$, Kolmogorov test). These distributions suggest that the tissue-derived exRNAs are at an equilibrium state of balanced supply and removal in serum.

### Nonuniform Presence of Different Fragments of a Long RNA in Serum.

The size distribution of exRNA suggested lack of full-length long RNA in serum (*SI Appendix*, Fig. S1), which raises the question of whether different parts of a long RNA had equal chances of being detected as exRNA. We used the KRAS oncogene as a test case for this question. In the 128 serum samples in this study (*SI Appendix*, Fig. S6 and Table S3), a total of 6,864 reads were uniquely mapped to KRAS, in which 5,576 reads (81.2%) were derived from the fourth exon (red curve, Fig. 2*D*), suggesting nonequal chances for different fragments of the KRAS transcripts to be present in serum (q value < $10^{\times }$^16^, Kolmogorov–Smirnov test for uniform distribution) (*SI Appendix*, Fig. S7). Next, we checked whether the abundance of Exon 4-derived exRNA was driven by a small number of serum samples. The Exon 4-derived exRNA was detected in the majority (78.1%) of the samples, whereas no other fragments of the KRAS were detected in more than 1/3 of the samples (green curve, Fig. 2*D*). In this case, the RNA fragments present in serum were nonuniform. Certain parts of KRAS mRNA had greater chances of presence in serum.

### exRNA Reflects Sex and Chronological Age.

**Fig. 3.**
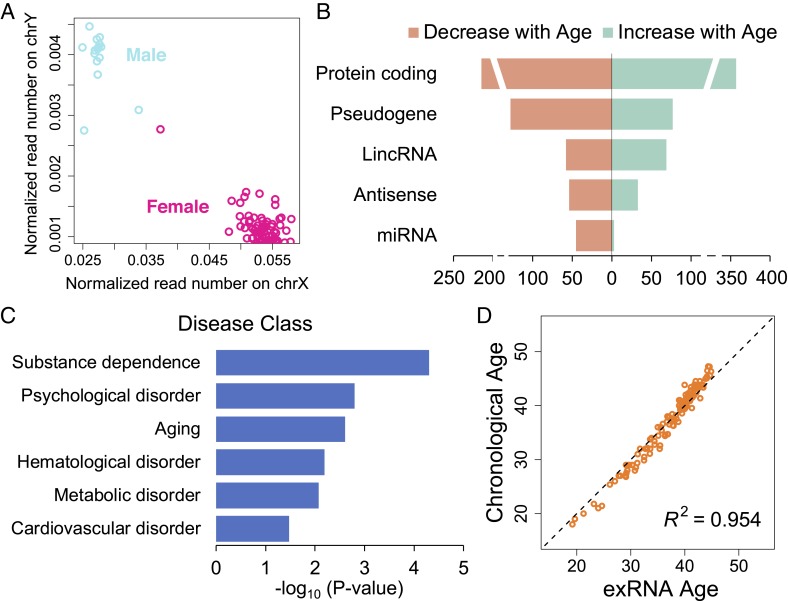
Correlations of exRNA expression with sex and age. (*A*) Scatter plot of normalized SILVER-seq reads mapped to X (*x* axis) and Y (y axis) chromosomes of every serum sample (circle). Male and female samples are colored in blue and red, respectively. (*B*) Numbers of exRNAs that are positively (green) and negatively (pink) correlated with age in each RNA type (row). (*C*) Disease classes (rows) that are associated with age-correlated exRNA genes; *x* axis, adjusted *P* value from association tests. (*D*) Scatter plot of exRNA age (*x* axis) and chronological age (*y* axis) for every sample (circle).

We asked whether exRNA correlates with sex and age, 2 most common physiological parameters. We applied SILVER-seq to analyze a total of 128 serum samples, which yielded, on average, 6.56 million uniquely mapped reads per sample (*SI Appendix*, Fig. S6 and Table S3). We plotted the normalized numbers of uniquely mapped SILVER-seq reads to the sex chromosomes of every serum sample (Fig. 3*A*). This completely separated the serum samples of males (blue) and females (red). This separation suggests a clear correspondence between patterns of exRNA expression and sex.

Next, we tested whether exRNA expression reflects a donor’s chronological age. A total of 1,149 exRNAs exhibited modest age-associated expression changes (*P* value < 0.01, F test, q values of these exRNAs range from 0.00002 to 0.41033), including mRNA- and noncoding RNA-derived exRNAs (Fig. 3 *B* and *C*). These age-correlated exRNAs were enriched in disease classes of substance dependence, psychological disorders, and aging (Benjamini adjusted *P* value = 0.015), as well as hematological, metabolic, and cardiovascular disorders [Benjamini-adjusted *P* value = 0.10; disease class enrichment analysis by DAVID (16)] (Fig. 3*C*). The exRNAs with the strongest positive correlations with age included VCAN, a proteoglycan involved in cell adhesion, MGAT4C, a glycosyltransferase required for proper lysosomal function, and TOR1AIP2, an endoplasmic reticulum membrane protein (*SI Appendix*, Fig. S8 *A*–*C*). The exRNAs with the strongest negative correlations with age included PRRG3, a vitamin K-dependent transmembrane protein, YBX1, a ribonucleoprotein (RNP) involved in microRNA processing and mRNA splicing, and FSTL3, a secreted glycoprotein that binds and inhibits Activin A and BMP2 signals (*SI Appendix*, Fig. S8 *D*–*F*). These top-ranked age-correlated exRNAs were derived from the mRNAs of secreted or transmembrane proteins that conjugate, bind, or modify glycans. Indeed, glycans have been nominated as a biomarker of biological age (17). These data suggest correlations between age-dependent circulating exRNA changes and age-dependent gene expression changes in various tissues.

We built a regression model using exRNA expression levels as covariates and age as the outcome. Hereafter, we denote the exRNA predicted age by this regression as exRNA age. The exRNA age exhibited a Pearson correlation of 0.986 with chronological age (Fig. 3*D*). Approximately 95.4% of the variation of chronological age was explained by exRNA age (*P* value < $10^{-32}$, F test). The exRNA age was within 2 y range of the chronological age for more than 90% of the samples. We tested sex, ethnicity, body mass index, smoking status, and drinking status as potential confounders. None of these factors exhibited any noticeable impact to the correlation between exRNA age and chronological age (all adjusted *P* values > 0.9). Taken together, exRNA age is predictive of chronological age. The correlation of SILVER-seq data and human physiology provided a baseline for us to move on to testing SILVER-seq’s predictive power to disease status.

### Similarity of Global exRNA Profiles between Cancer and Normal Sera.

**Fig. 4.**
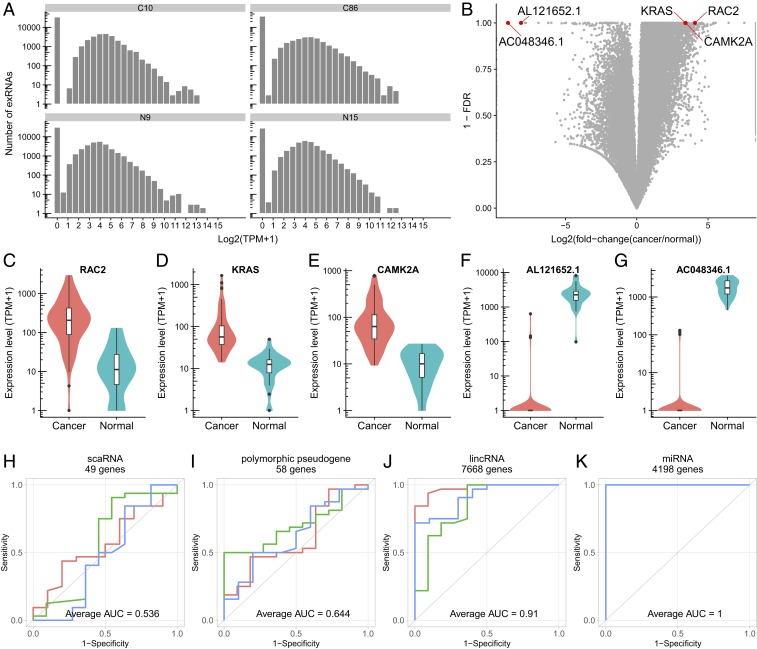
The exRNA expression in cancer and normal serum samples. (*A*) Distribution of exRNA expression levels of every gene in the human genome (60,675 genes in total, hg38) in 2 representative cancer samples (C10, C86) and 2 representative normal samples (N9, N15). See *SI Appendix*, Fig. S9 for all other samples. (*B*) Volcano plot of log fold change (cancer/normal) (*x* axis) and FDR (*y* axis) for all exRNAs (dots). (*C*–*G*) Expression levels of (*C*) RAC2, (*D*) KRAS, (*E*) CAMK2A, and (*F*) AL121652.1 and (*G*) AC048346.1 exRNA in cancer (red) and normal serum (blue). (*H*–*K*) receiver operating characteristic (ROC) curves of classification results based on (*H*) scaRNA, (*I*) polymorphic pseudogene, (*J*) lincRNA, and (*K*) miRNA, based on 3-fold cross-validations (red, green, blue).

We tested whether the overall distributions of exRNAs were different between cancer and normal sera. Our SILVER-seq datasets included 96 serum samples of breast cancer patients (cancer samples) (*SI Appendix*, Table S4) and 32 serum samples from other donors who did not have self-reported disease (normal samples) (*SI Appendix*, Table S3). TPM were calculated for each exRNA and used as the surrogate metric for the expression level of the exRNA. The distributions of TPM exhibited little difference between any 2 cancer samples or between a cancer sample and a normal sample (Fig. 4*A* and *SI Appendix*, Fig. S9). Thus, every sample contains a similar proportion of highly expressed exRNAs, regardless of the threshold for calling highly expressed exRNAs.

### Differentially Expressed exRNAs between Cancer and Normal Donors.

To test for differential expression of exRNAs between the serum samples collected from cancer and normal donors, we computed the fold change and false discovery rate (FDR) for every exRNA (Fig. 4*B*). Regardless of the FDR threshold, there were more exRNAs with higher expression in cancer (cancer-upregulated) than those with lower expression in cancer (cancer-downregulated) as compared to normal samples (Fig. 4*B*). The cancer-upregulated exRNAs that were also most frequently detected among the cancer samples that came from RAC2, KRAS, and CAMK2A (Fig. 4 *C*–*E*). RAC2 and KRAS are 2 members of the Ras proto-oncogene superfamily, associated with breast cancer tumorigenesis and metastasis (18) (*SI Appendix*, Fig. S10). The upregulation of calcium-dependent protein kinase CAMK2A likely reflects perturbed calcium homeostasis, a hallmark of cancer (19). The cancer-downregulated exRNAs with the highest recurrence in normal samples were long intergenic noncoding RNA (lincRNA) AL121652.1 and pseudogene RNA AC048346.1 (Fig. 4 *F* and *G*). Thus, the top-ranked exRNAs came from both coding and noncoding RNAs.

### The Different Capacity of Different RNA Types in Differentiating Cancer and Normal Serum Samples.

We asked whether different types of RNAs exhibit the same power of differentiating cancer and normal samples. To establish a baseline, we did a principal component analysis (PCA) using exRNAs of all known genes (60,675 genes, hg38) (*SI Appendix*, Fig. S11*A*). Cancer and noncancer samples were not distinguishable by the first principal component (PC1), but they exhibited some extent of separation on the second principal component (PC2) (*SI Appendix*, Fig. S11*A*). These data suggest not only large sample-to-sample variations, but also the possible separation of cancer and noncancer samples by some subspaces (subsets of genes). This global feature is not sensitive to the number of exRNAs used for PCA analysis (*SI Appendix*, Fig. S11 *B* and *C*).

We proceeded to test whether the degrees of cancer-normal separation are similar across different types of RNAs. To this end, we did a PCA analysis with each type of RNA. Three classes of RNA types emerged based on the capacity of their principal components to explain cancer-normal differences. The first class failed to separate cancer and normal samples by either PC1 or PC2 (*SI Appendix*, Fig. S12*A*). The second class exhibited some differentiation capability in PC2 but not in PC1 (*SI Appendix*, Fig. S12*B*). This class, which included protein-coding transcripts, processed pseudogenes, lincRNAs, and others, reflects the baseline (*SI Appendix*, Fig. S11) in that, although cancer-normal differences contributed to explain sample difference, it was not the major contributor to sample variations (PC1). The third class was able to differentiate cancer and normal samples in both PC1 and PC2. This class included miRNA, mitochondrial transfer RNA (Mt_tRNA), ribosomal RNA (rRNA), and other noncoding RNA (misc_RNA). With the third class, the major contributor to sample variation is the cancer/noncancer status. Taken together, cancer and noncancer samples are well separated in some subspaces, including the subspaces defined by the miRNAs and Mt_tRNAs.

### Classifying Cancer and Normal Samples without Preselecting Differentially Expressed exRNAs.

We asked to what extent the cancer and normal sera could be correctly classified by SILVER-seq data. First, we used the 1,719 differentially expressed exRNAs (|log2(fold change)|>2 and FDR < 0.05) as the feature set. All cancer and normal serum samples were correctly classified by a supporting vector machine (SVM) with 100 cross-validations (average area under curve [AUC] = 1.0).

To avoid overfitting, we asked whether sera from cancer patients and normal donors can be classified without using differentially expressed exRNAs as features. To this end, we used all of the annotated genes in the human genome. The human genes were classified by their RNA type (also called biotype) into protein coding, pseudogene, and noncoding genes, which were further categorized into 17 subtypes, including antisense, lincRNA, miRNA, and small nuclear RNA (snRNA) (20). We used all of the RNAs of each biotype as a feature set to carry out classification with random forest (*SI Appendix*, Fig. S13) and SVM (*SI Appendix*, Fig. S14). The different RNA types exhibited different classification performances. Several transcript categories, including small Cajal body-specific RNA (scaRNA) and polymorphic pseudogene, failed to classify cancer and normal samples (Fig. 4 *H* and *I* and *SI Appendix*, Figs. S13 and S14). On the other hand, using lincRNA and miRNA as feature sets improved classification performances (Fig. 4 *J* and *K*). In particular, miRNAs as a feature set nearly perfectly classified cancer and normal samples (Fig. 4*K*). These classification results independent of preselected differentially expressed exRNAs suggest that the cancer-normal differences are an intrinsic characteristic of the circulating extracellular transcriptome.

### Difference between Patients with and without Cancer Recurrence.

We tested whether there is any difference in exRNA expression that may correspond to cancer recurrence. The 96 analyzed serum samples were collected from breast cancer patients during a 5-y follow-up starting from their chemotherapy start date. Among them, 28 and 68 samples were collected from patients who developed and did not develop recurring cancer, respectively, in the 5-y follow-up; these 2 groups of serum samples will be referred to as recurrence and nonrecurrence samples, respectively. No exRNA was called as differentially expressed at the significance level of FDR = 0.1, suggesting that the difference between recurrence and nonrecurrence samples is more obscure than the difference between cancer and normal serum samples. Nevertheless, based on 2,230 exRNAs that exhibited fold changes of 2 or greater, recurrence and nonrecurrence samples could be accurately classified (AUC > 0.999, 100 cross-validations).

**Fig. 5.**
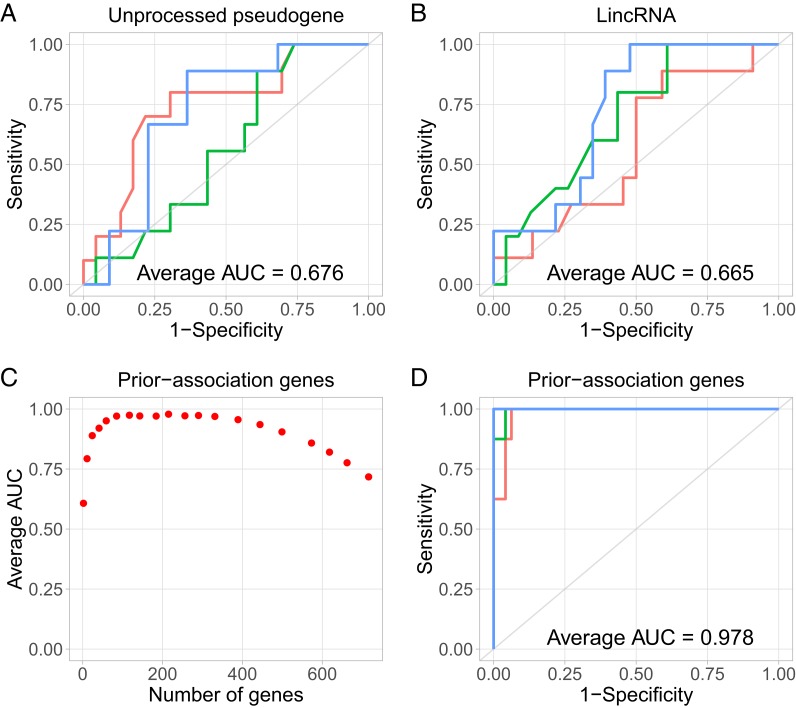
Classification of serum samples from patients with or without cancer recurrence. (*A* and *B*) Representative ROC curves from cross-validations, using all of the genes of each RNA type as features, including (*A*) unprocessed pseudogenes and (*B*) lincRNAs. (*C* and *D*) Classifications based on prior-association genes. (*C*) Average AUC of 100 cross-validations (*y* axis) based each number of prior-association genes used as features (*x* axis). (*D*) Representative ROC curves from 100 cross-validation based on 215 prior-association genes as features.

To avoid overfitting, we proceeded with classifications without using differentially expressed exRNAs. First, we used all of the genes of each RNA biotype, including mRNA, lincRNA, miRNA, and others (20). As expected, the cross-validation AUCs were close to 0.5 for most of the RNA biotypes (*SI Appendix*, Figs. S15 and S16), consistent with the idea that recurrence and nonrecurrence samples were less separated than cancer and normal samples. Nevertheless, classifications based on several RNA biotypes, including unprocessed pseudogene (21) and lincRNA, resulted in better AUCs than random guesses in cross-validations (Fig. 5 *A* and *B*). These data suggest a moderate separation of recurrence and nonrecurrence samples.

Next, we compiled a list of 750 genes that were associated with breast cancer by prior literature (prior-association genes) (*SI Appendix*, Table S5). This mixed group of genes were associated with breast cancer in many ways, including genotype–disease association and the associations of gene expression in cancer biopsy and cancer subtypes, grades, or prognoses. When this mixed bag of literature-derived genes was used as the feature set, an SVM classifier was able to better distinguish recurrence and nonrecurrence samples (average AUC = 0.720) than using the genes of any biotype (maximum average AUC = 0.696) (*SI Appendix*, Figs. S15 and S16). These data suggest that the exRNA expression of the prior-association genes was correlated with recurrence status, even though the prior association was not discovered using exRNA expression.

When we varied the number of prior-association genes in the feature set, the classification performance peaked between 150 and 400 genes and decreased when either fewer or more prior-association genes were used (Fig. 5*C*). For example, using 215 prior-association genes as the feature set, the average AUC reached 0.978 in 100 cross-validations (Fig. 5*D*). These data support that exRNA expression contains information about the recurrence status in breast cancer patients. However, such information likely resides in a combination of exRNAs rather than in any individual exRNA.

## Discussion

### Breaking the Bottleneck of Input Volume for Serum exRNA Sequencing.

The standard input volume for serum exRNA sequencing analysis is several milliliters (4, 22, 23). Smaller input volume would result in too little RNA after the RNA extraction step. To overcome this bottleneck, we recognized that an enabling step in the transformation from bulk-cell RNA sequencing to single-cell RNA sequencing was to skip RNA extraction and directly carry out complementary DNA (cDNA) synthesis in cell lysis solution (9, 24). This idea prompted us to test exRNA sequencing from ultralow input of serum by cDNA synthesis in serum lysis solution without RNA extraction, which became the central ideal of SILVER-seq.

Even with plenty of (milliliters) serum as input, large measurement variations of exRNA from sequencing have been reported (22, 25). Despite these variations, the relative abundances of different exRNAs were, to some extent, comparable across different exRNA sequencing methods (22, 25). Importantly, the correlation between 2 standard RNA-seq datasets was comparable to that between a SILVER-seq and a standard RNA-seq, as well as that between 2 SILVER-seq datasets. These results indicate that SILVER-seq effectively restrained additional measurement variations due to the decrease of input volume.

We suspect that the biological variation in exRNA content between 2 serum droplets is primarily attributable to the different composition of exRNA carriers. These carriers include extracellular vesicles (EVs), RNPs, lipoprotein complexes (22), nonmembranous nanoparticles (exomeres) (26), and other to-be-identified types. It would require future technology developments to quantify the variability of cargo composition between 2 serum samples, especially between 2 small-volume samples.

### Fragments of Long RNAs in Human Serum.

Most previous analyses focused on small RNAs (4, 22, 25). However, up to 55% of serum-extracted RNA sequences could not be aligned to small RNAs (22), begging the question of what other RNAs are present in human sera. SILVER-seq revealed large amounts of long RNA fragments in human sera. These fragments were typically 200 nt or smaller in length. They were derived from mRNAs, lncRNAs, and pseudogene RNAs. The host RNAs of these fragments could exhibit tissue specificity in expression. As a result, the majority of tissue-specific RNAs, including brain-specific RNAs, were detectable in human serum as fragments. Some of these fragments derived from cancer-related genes, including KRAS, were among the most upregulated exRNAs in cancer patients as compared to normal donors. These data suggest the value of including RNA fragments in future liquid biopsy-based IVD research.

### Serum exRNA Reflects Sex and Age.

We hypothesized exRNA in serum reflects differences based on a donor’s age and sex. However, a recent analysis reported a counterintuitive observation that the sex-associated exRNAs in human serum were not expressed from the sex chromosomes (22). There is, as yet, no literature on testing the association of any biofluid exRNA to age. This study reported a strong association of sex chromosome-derived exRNAs with donor’s sex, and a strong association of several hundred exRNAs to donor’s chronological ages (Fig. 3). Furthermore, the age-associated exRNAs overlapped with the previously identified genes with age-dependent expression in various tissues and were enriched for the genes associated with age-related disorders. These data support using exRNA to monitor human physiology.

This study only analyzed donors between 18 y and 48 y old. The identified age-associated exRNAs are probably specific to this age group and cannot be extrapolated to older ages. For example, the genes involved in substance dependence and psychological disorders (Fig. 3*C*) are primarily expressed in brain. Gene expression changes in adolescent and adult brains are associated with different vulnerabilities for substance addition (27–30). Thus, this subset of age-related exRNAs may have reflected the changes of the brain between early and middle adulthood.

### The Differentiating Power of miRNAs and Mt_tRNAs to Classify Breast Cancer Patients and Normal Donors.

A common practice to avoid overfitting is to subject the biomarkers developed from one patient cohort to validation in another cohort. However, there is only one cohort in this study. To minimize overfitting in this scenario, we did not use the common practice of using differentially expressed exRNAs as features for classification. Instead, we used the entire list of genes of each gene category (protein coding, lincRNA, antisense, miRNA, etc.) as a feature set to classification. This approach tested whether the exRNAs of each gene category as a whole contain any information on the disease status. Interestingly, miRNAs and Mt_tRNAs exhibited the largest differentiating powers to classify breast cancer patients and normal donors. These data expanded the previously reported clinical variables that correlate with serum/plasma miRNAs (6, 7). These data also nominate serum extracellular Mt_tRNAs as another prominent class of molecules in developing clinically relevant biomarkers.

### Limitations of This Study.

Breast cancers include several molecular subtypes. This study included 10, 48, 12, and 26 samples from Her2-enriched, luminal A or normal-like, luminal B, and triple-negative subtypes, respectively (*SI Appendix*, Table S4). The top 100 exRNAs that were most correlated with subtype differences (ANOVA, q value ranges from 0.055 to 0.999) included ODC1 (31), RBP3 (32), and WIF1 (33) that were also differentially expressed in the tissue biopsies between these subtypes (*SI Appendix*, Fig. S17). Thus, exRNA expression may reflect the differences between different subtypes of breast cancer. However, the small number of samples in each subtype is insufficient to assess the significance of such correlations.

This study did not rule out all possible confounding factors that may contribute the separation of cancer and normal samples. Most of the serum samples from cancer patients were collected during or after chemotherapy (*SI Appendix*, Table S4). Thus, this study cannot separate chemotherapy-induced changes from cancer-induced changes. However, the consistent upregulation of RAC2 and KRAS exRNAs in serum and mRNAs in tissue in breast cancer patients as compared to normal donors, together with the known roles of these 2 members of the Ras proto-oncogene superfamily in breast cancer etiology, suggest that a subset of the observed serum exRNA expression changes relate to the disease rather than the treatments. Future studies that control for treatment status and cancer subtypes are needed, preferably as double-blind prospective trials.

## Materials and Methods

### Human Serum Samples.

Obtaining and analysis of deidentified human sera has been approved by University of California San Diego Human Research Protections Program.

### Analysis of Sizes of exRNAs in Serum.

A total of 9 serum samples of 1-mL volume were analyzed (samples 1 to 9, *SI Appendix*, Fig. S1). RNA of each sample was purified by one of the 3 kits, namely, exoRNeasy Serum/Plasma Midi Kit (QIAGEN), TRIzol LS Reagent (Invitrogen), or Plasma/Serum RNA Purification Kit (NORGEN). The RNA extracted with the NORGEN kit was treated with RNase-Free DNase I (QIAGEN) and RNeasy MinElute Cleanup Kit (QIAGEN) according to manufacturer’s instruction. Another serum sample of 200-μL volume was also analyzed (sample 10; *SI Appendix*, Fig. S1). RNA from this sample was purified with the QIAzol (QIAGEN) kit. Extracted RNA was stored at −80 °C until use. RNA sizes were analyzed by the bioanalyzer RNA pico chip (Agilent).

### Construction of SILVER-seq Sequencing Libraries.

The starting volume of each serum sample was between 3 μL and 7 μL. Any serum sample of volume smaller than 7 μL was supplemented with Ultrapure water to reach a total volume of 7 μL. EVs were lysed, and RNPs were disassociated by mixing the sample with 1.7 μL of 11.5 mM DTT solution, 0.5 μL of 40 U/μL RNase inhibitor, and 2.8 μL of lysis buffer consisting of 10 mM Tris-HCl, 0.2% w/v SDS solution, and 4% w/v Nonidet P-40. First- and second-strand cDNA syntheses were carried out as follows (*SI Appendix*, Fig. S2) (https://www.genemo.com/technology/silver-seq). The resulting material from the previous step was incubated with a mix of random hexamer and oligo-dT primers at 70 °C for 2 min, and incubated with temperature-sensitive double-strand DNase (HL-dsDNase) at 37 °C for 10 min, then at 65 °C for 5 min for enzyme deactivation, and subsequently incubated with reverse transcriptase at 25 °C for 5 min followed by 40 °C for 30 min and 70 °C for 10 min. The resulting material was incubated with DNA polymerase and template-switching oligo at 25 °C for 15 min, at 37 °C for 15 min, and then 70 °C for 10 min and subjected to end repair, adaptor ligation, size selection, amplification, and rRNA sequence depletion (https://www.genemo.com/technology/silver-seq). The product library was quantified with Qubit (Invitrogen), and measured by Bioanalyzer (Agilent) for size distribution.

### Alignment to Reference Genome.

STAR (STAR_2.5.1b, default parameters) was used to align SILVER-seq and RNA-seq reads to the reference genome (hg38). Uniquely aligned reads were used together with the gene annotation file (Hg38/Ensembl) as input files to HTSeq-count (version 0.9.1) to count the number of reads per gene, which was subsequently transformed in TPM.

### Association Analysis of exRNA and Chronological Age.

F test was used to test the correlation of the TPM of every exRNA with chronological age. The F test-derived *P* values were provided to the R package {qvalue} to calculate q values. The chronological age and the top 500 exRNAs with the largest Pearson correlation with age were given to the R package {glmnet} to fit a linear regression with elastic net regularization.

### Calculating the Frequency of Detecting an exRNA.

An exRNA is called detected in a sample at the threshold of TPM > 5. The frequency of detecting an exRNA among the samples was calculated as the proportion of samples in which this exRNA was detected.

### Gene Categories and RNA Types.

The gene categories as defined by Ensembl were used in PCA and classification analyses. Ensembl categorized genes by their RNA types, also called RNA biotypes. A total of 23 gene categories contained at least 10 genes per category, which included protein coding, lincRNA, miRNA, snRNA, and other biotypes.

### Classification Analysis.

Classification of cancer samples including both recurrence and nonrecurrence samples and noncancer samples was carried out with both random forest and linear kernel SVM using R package {mlr} (34). Each feature set was defined as all of the exRNAs of each gene category. The log-transformed TPMs (log2(TPM+1)) of every exRNA were given as the input data. Threefold cross-validations were carried out unless otherwise stated.

Classification of recurrence and nonrecurrence cancer samples were carried out using the same procedure as that used for classification of cancer and noncancer samples. In addition, all analyses were repeated using the prior-association genes (*SI Appendix*, Table S5) as a feature set.

## Supplementary Material

Supplementary File
