# Supplementary material for: Extracellular RNA in a single droplet of human serum reflects physiologic and disease states
Source: Proc Natl Acad Sci U S A. 2019 Sep 3;116(38):19200–8. doi: 10.1073/pnas.1908252116 (PMC6754586; doi:10.1073/pnas.1908252116)
Supplement: Supplementary File [file pnas.1908252116.sapp.pdf]

PNAS
www.pnas.org

Supplementary Information for

Extracellular RNA in a single droplet of human serum reflects physiologic and disease states

Zixu Zhou, Qiuyang Wu, Zhangming Yan, Haizi Zheng, Chienju Chen, Yuan Liu, Zhijie Qi, Riccardo Calandrelli, Zhen Chen, Shu Chien, H. Irene Su and Sheng Zhong

Shu Chien, H. Irene Su, and Sheng Zhong
Email: shuchien@ucsd.edu, hisu@ucsd.edu and szhong@ucsd.edu

This PDF file includes:

Figs. S1 to S17
Tables S1 to S5
References for SI reference citations

www.pnas.org/cgi/doi/10.1073/pnas.1908252116

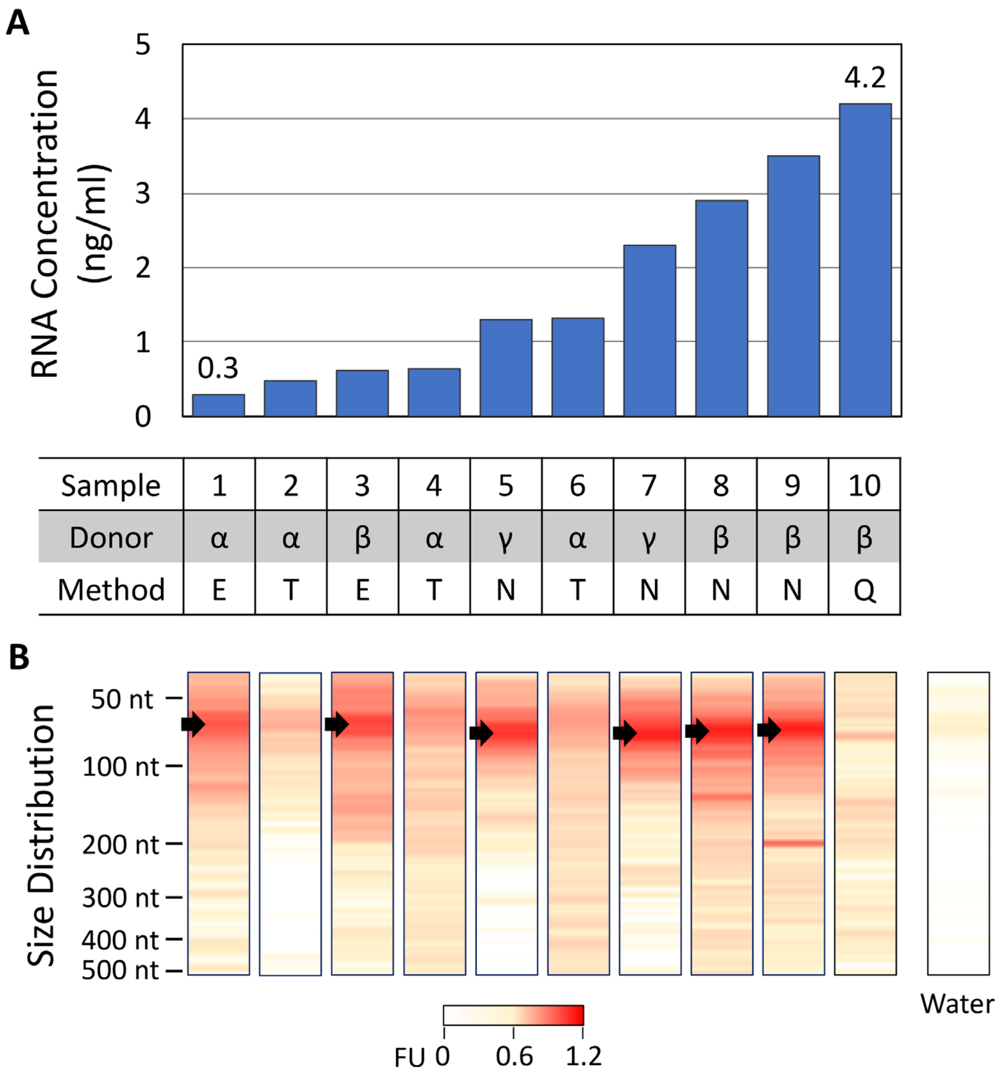

Fig. S1. Concentrations and lengths of exRNA in human serum. (A) Measured concentrations of exRNA (y axis) in ten serum samples (columns) from three donors (indexed by α, β, γ). The exRNA of each sample was purified by one of the four commercially available methods (indexed by E, N, Q, T). E: exoRNeasy by QIAGEN, N: NORGEN RNA purification kit, Q: QIAzol by QIAGEN, T: TRIzol^{TM} LS by ThermoFisher. (B) Length distributions of exRNA. The relative densities in arbitrary fluorescent unit (FU, scale bar) are plotted against the length separated exRNA (nucleotides (nt), y axis) in each sample (lane). Arrows: density peaks. As a control, 1 μL Ultrapure water was given as input to bioanalyzer (Water lane), which yielded smaller than 0.2 FU at any size. Nearly all detected exRNA are within the size range of 20 to 200 nt (FU > 0.2).

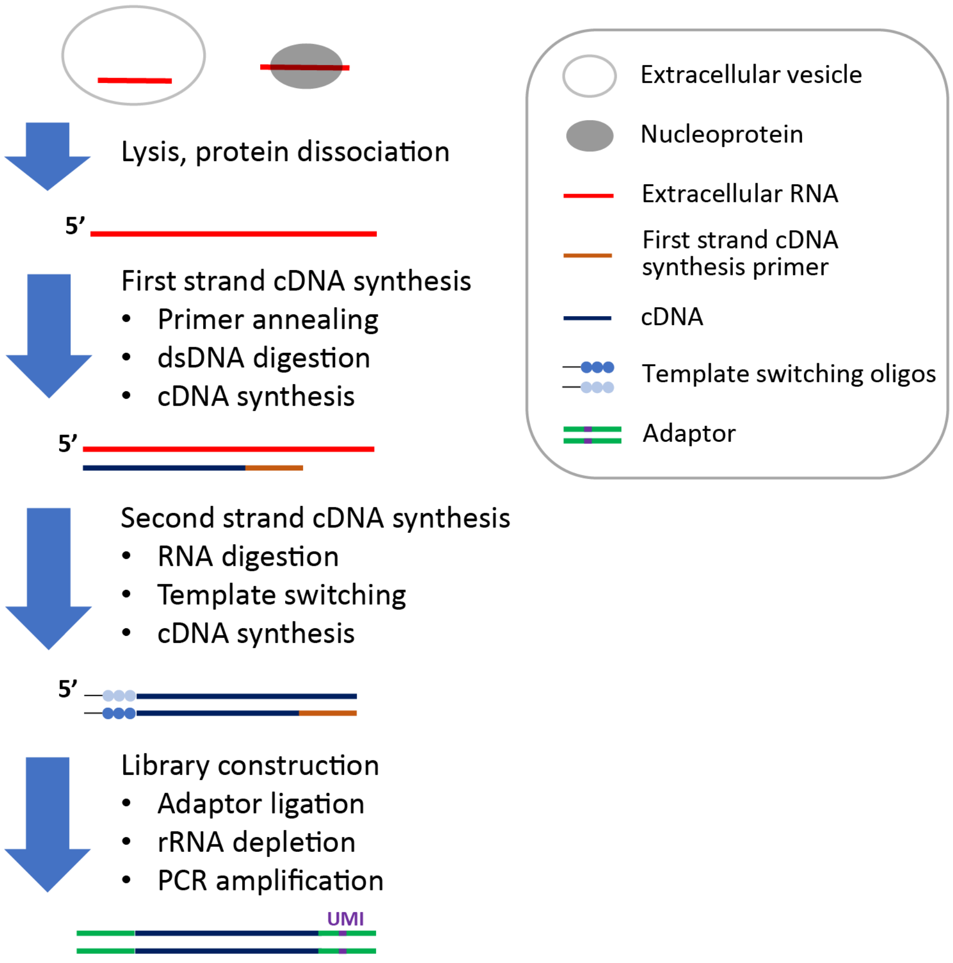

Fig. S2. Flowchart of the SILVER-seq pipeline. UMI: Unique molecular identifiers.

Volume (µL)	3	3	3	3	3	3	3	3	3	7	7	7	7	7	7
Donor #	2	2	2	2	3	3	3	3	3	2	2	3	3	3	3
Aliquot #	1	2	3	4	5	6	7	8	9	10	11	12	13	14	15

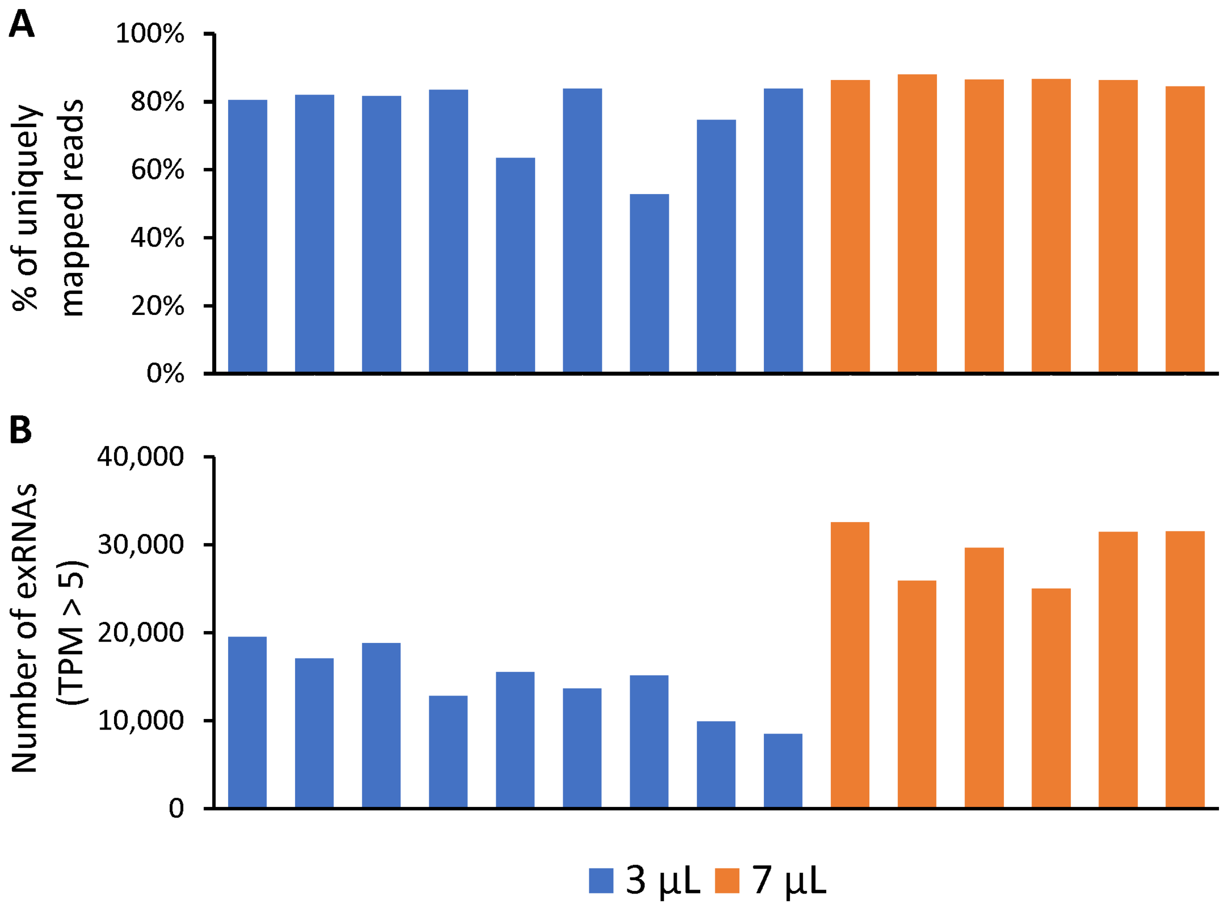

Fig. S3. Comparison of input volumes with serum from two additional donors. (A) Percentage of uniquely mapped reads (y axis) for each library. (B) Number of exRNAs with 5 or more Transcripts Per Million (TPM) in each library (column).

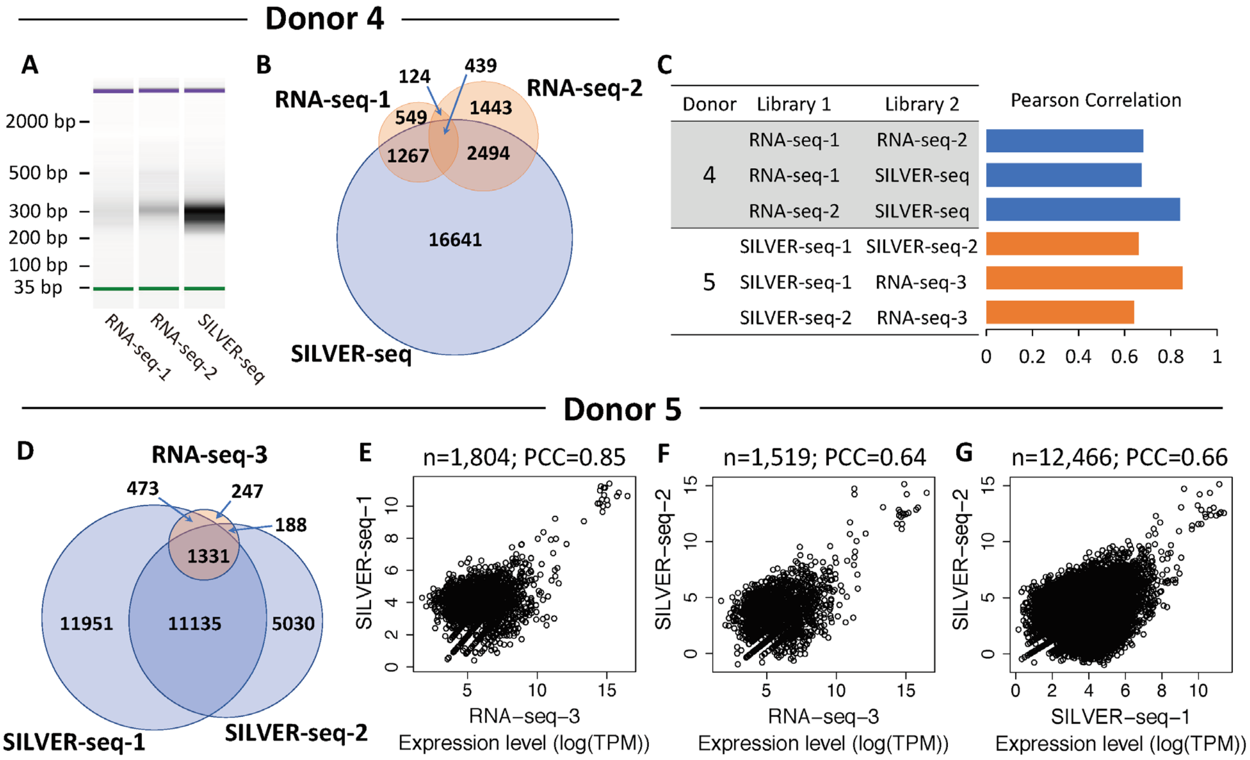

Fig. S4. Comparison of SILVER-seq and standard RNA-seq. (A-B) Comparison of standard RNA-seq libraries (RNA-seq-1, RNA-seq-2) and a SILVER-seq library generated from different aliquots of the same serum sample (Donor 4). (A) Bioanalyzer derived size distributions of the final sequencing libraries. (B) Venn diagram of the numbers of detected exRNA genes from the three libraries. (C) Pairwise Pearson correlation coefficient (PCC) of measured exRNA levels between two sequencing libraries, based on serum aliquots from Donor 4 (blue bars) and 5 (orange bars). (D-G) Variability of SILVER-seq measurements. (D) Venn diagram of the numbers of detected exRNA genes from two SILVER-seq libraries (SILVER-seq-1, SILVER-seq-2) and a standard RNA-seq library (RNA-seq-3). (E-G) Scatter plots of exRNAs (dots) with expression levels (log(TPM)) derived from RNA-seq-3 (x axis) and SILVER-seq-1 (y axis) (E), RNA-seq-3 and SILVER-seq-2 (F), as well as SILVER-seq-1 and SILVER-seq-2 (G).

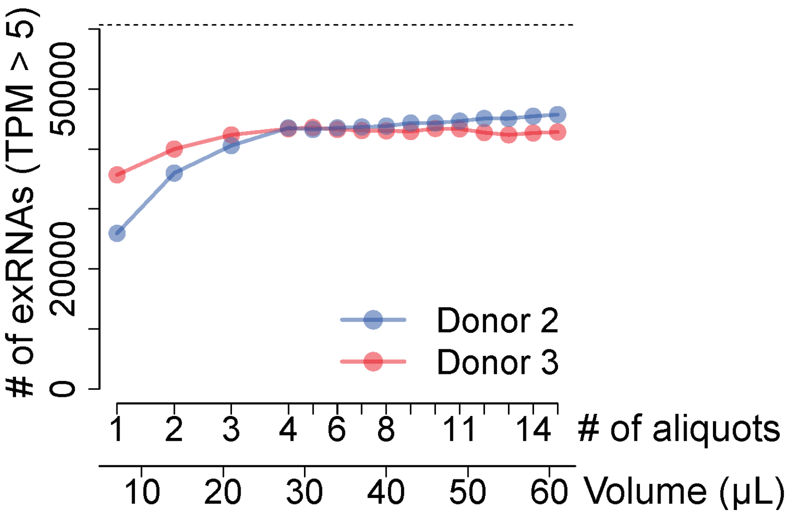

Fig. S5. Saturation analysis. The number of exRNAs (y axis) detected from the pooled sequencing data of the number of aliquots (# of aliquots, x axis). Volume: the cumulative volume of the corresponding number of aliquots.

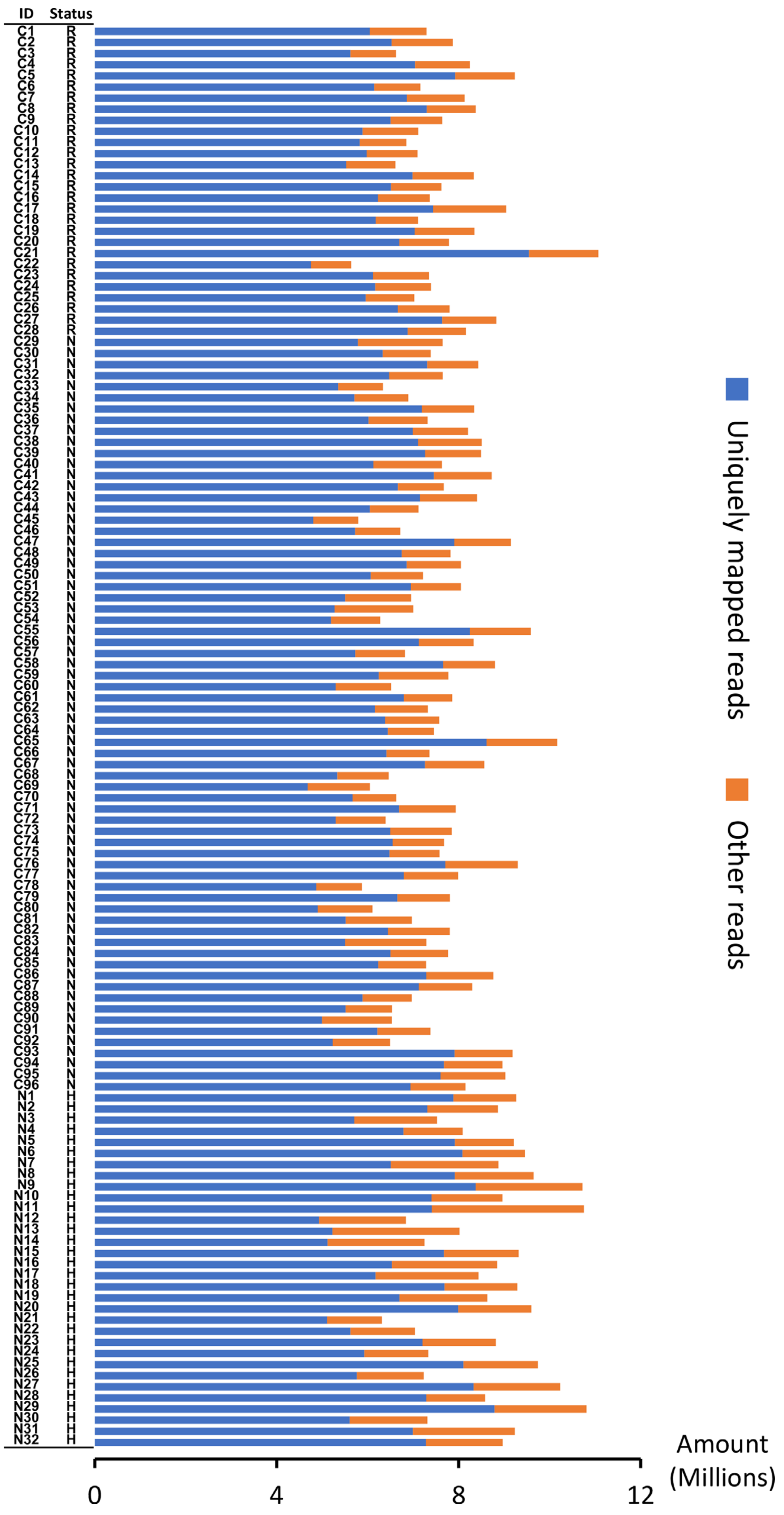

Fig. S6. Summary of 128 serum samples by sequencing depth (x axis). Status R: cancer with recurrence; N: cancer without recurrence, H: normal. Also see Table S3.

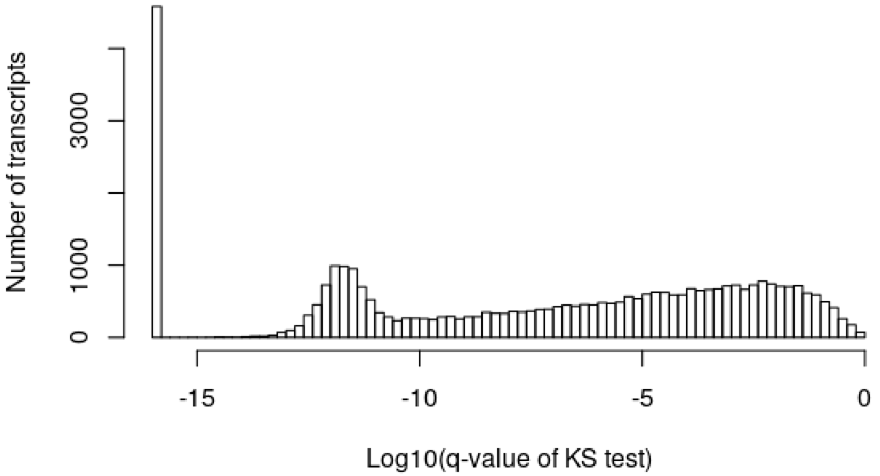

Fig. S7. Histogram of q-values derived from KS tests for uniform distribution. Each full-length transcript that is longer than 500 nt and covered by at least 100 sequencing reads was subjected to a KS test. The null hypothesis is that the sequencing reads are uniformly distributed across the full-length transcript. The alternative hypothesis is that the sequencing reads are not uniformly distributed. The KS test q-value indicates the degree of deviation of the reads from uniformly covering this full-length transcript.

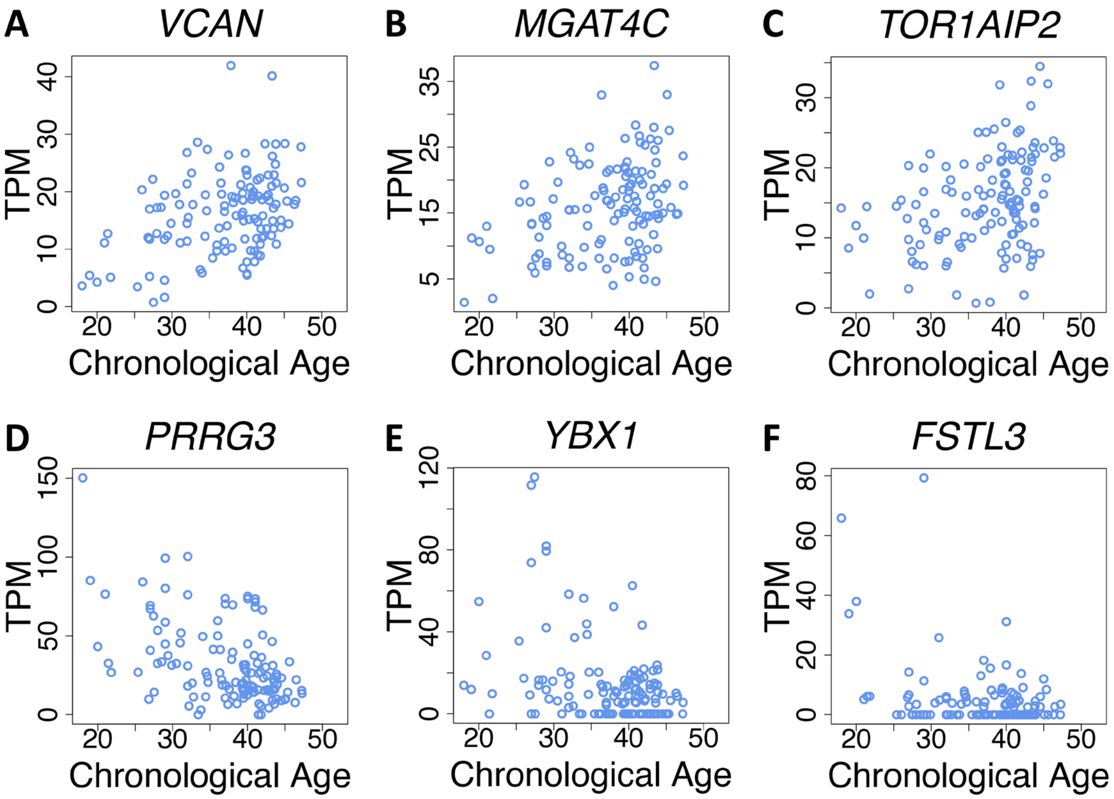

Fig. S8. Age associated exRNAs. exRNAs with expression levels (y axis) positively (A-C) and negatively (D-F) correlated with chronological age (x axis).

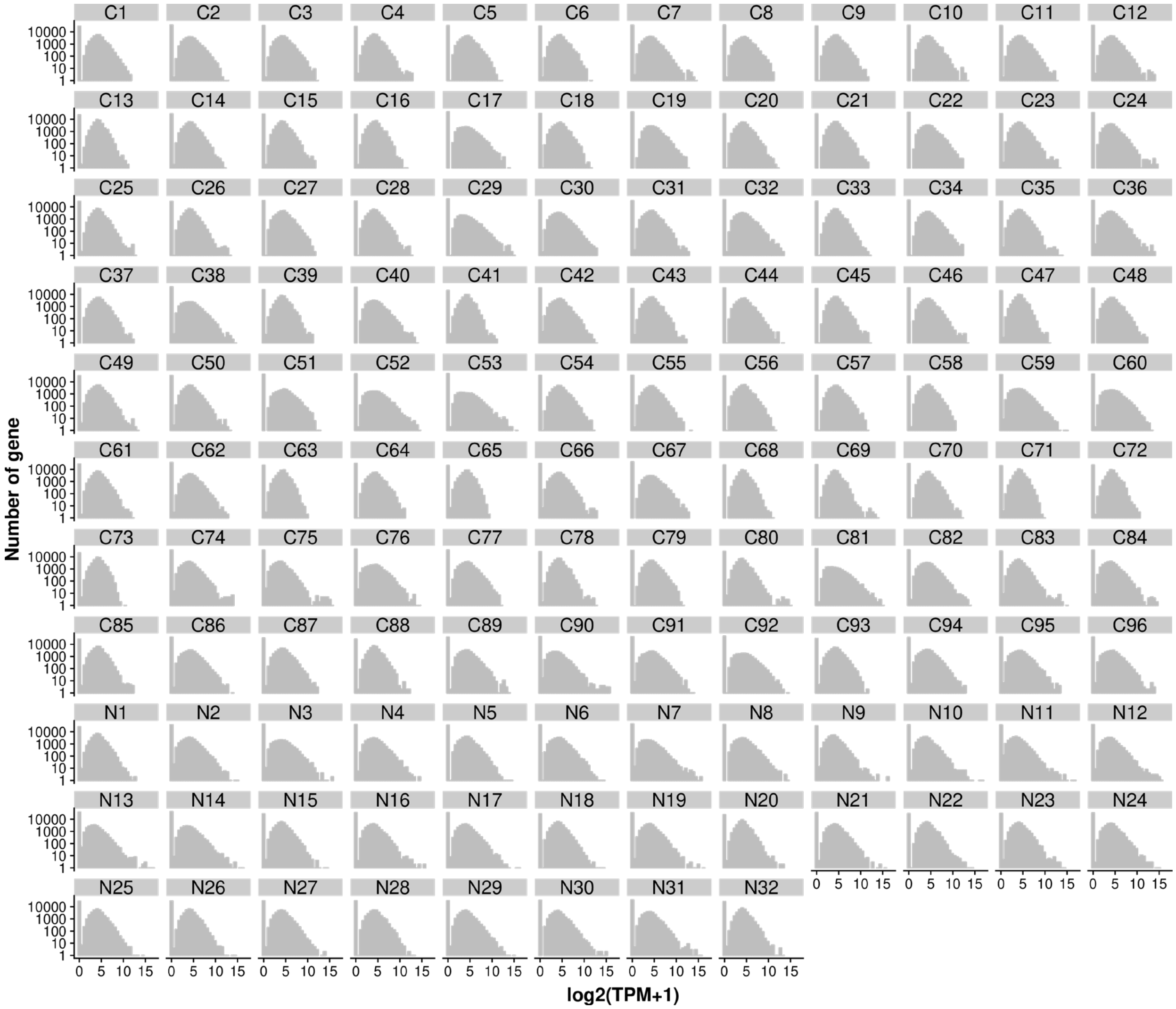

Fig. S9. Histograms exRNA expression level (Log2(TPM+1), x axis) in every sample. C1 – C96: cancer samples. N1 – N32: normal samples.

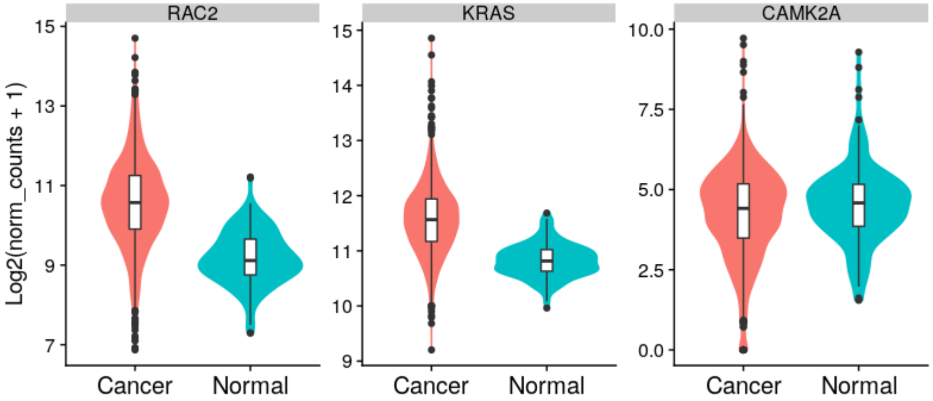

Fig. S10. Comparison of expression levels between breast cancer and normal breast. The RNA-seq data of 1,099 breast cancer tissue samples were generated by TCGA (red) and 178 normal breast tissues were generated by GTEx (blue) and were uniformly processed by UCSC's Xena platform. The normalized read counts (y axis) produced by Xena removed data processing differences and minimized batch efforts.

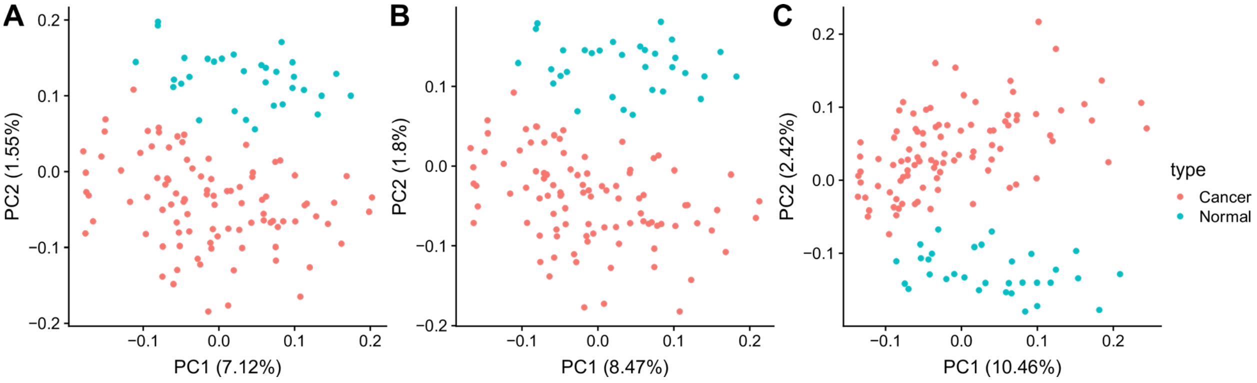

Fig. S11. Sensitivity of the PCA analysis to the choice of genes. (A) PCA with all genes (60,675 annotated genes, Hg38). (B) PCA analyses with the exRNAs that were detected in either 25% or more cancer samples or 25% or more normal samples (25%-recurring exRNAs). (C) PCA analyses with the exRNAs that were detected in either 50% or more cancer samples or 50% or more normal samples (50%-recurring exRNAs). Cancer and normal samples remained mixed in PC1 but somewhat separated in PC2. Red: cancer samples. Blue: normal samples.

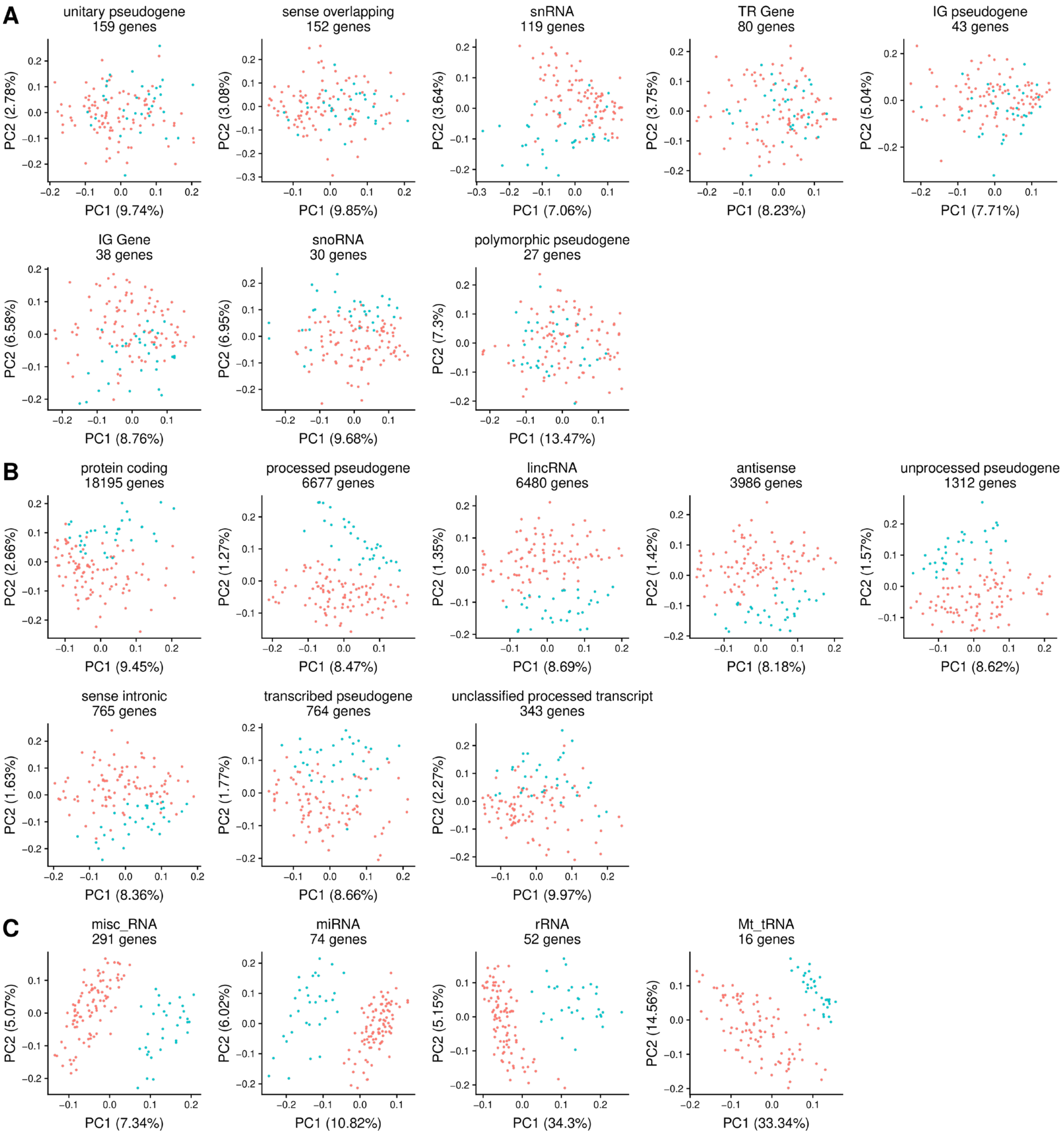

Fig. S12. PCA plots by RNA type. The genes of each RNA type (plot title) that were detected in either 25% or more cancer samples or 25% or more normal samples were used for PCA analysis. (A-C) The RNA types that poorly (A), moderately (B), and clearly separated (C) cancer (red) and normal samples (blue) by the first two principal components.

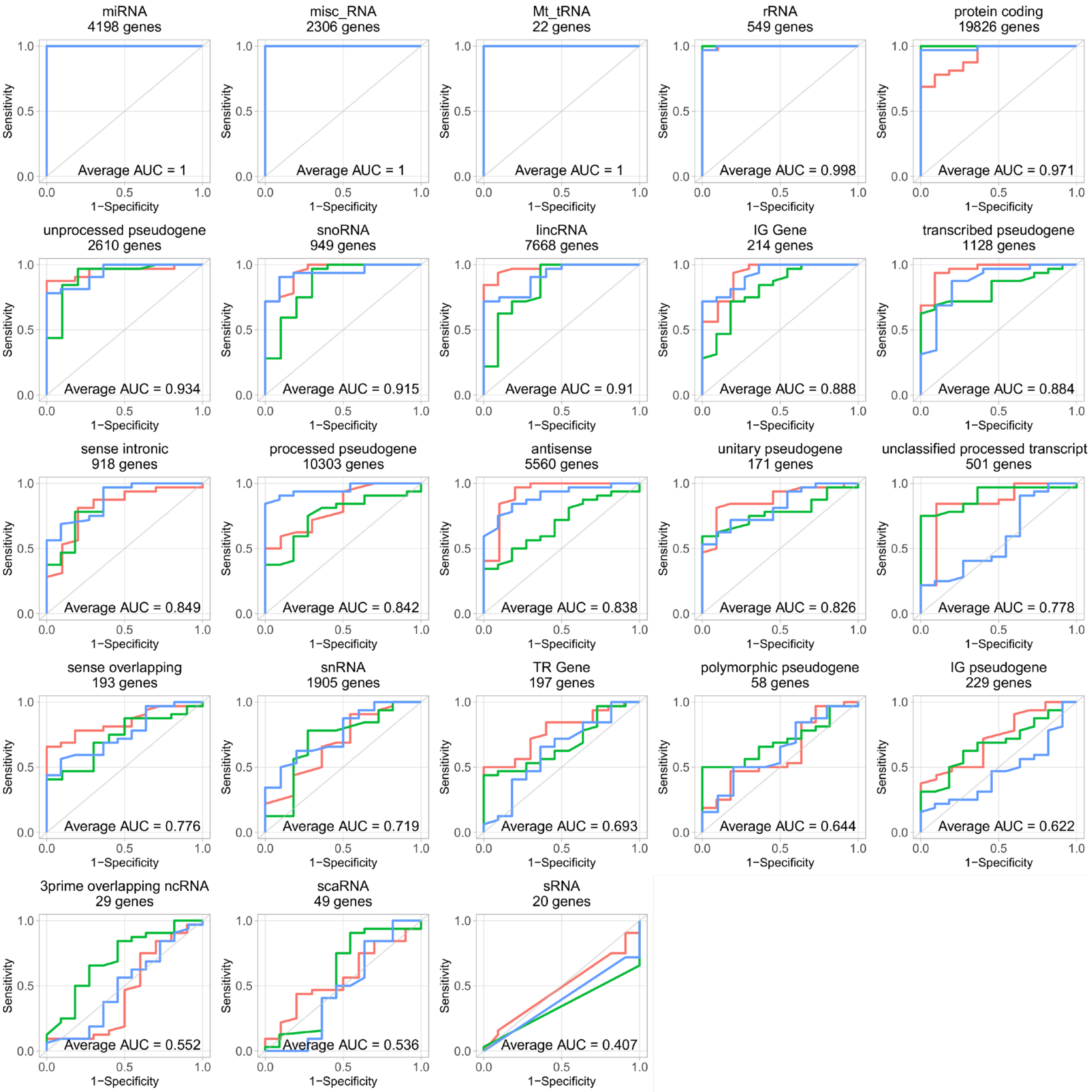

Fig. S13. Classification of cancer and normal serums by random forest. All the genes of each RNA type (panel title) were used as the features. Three ROC curves were generated from 3-fold cross validations.

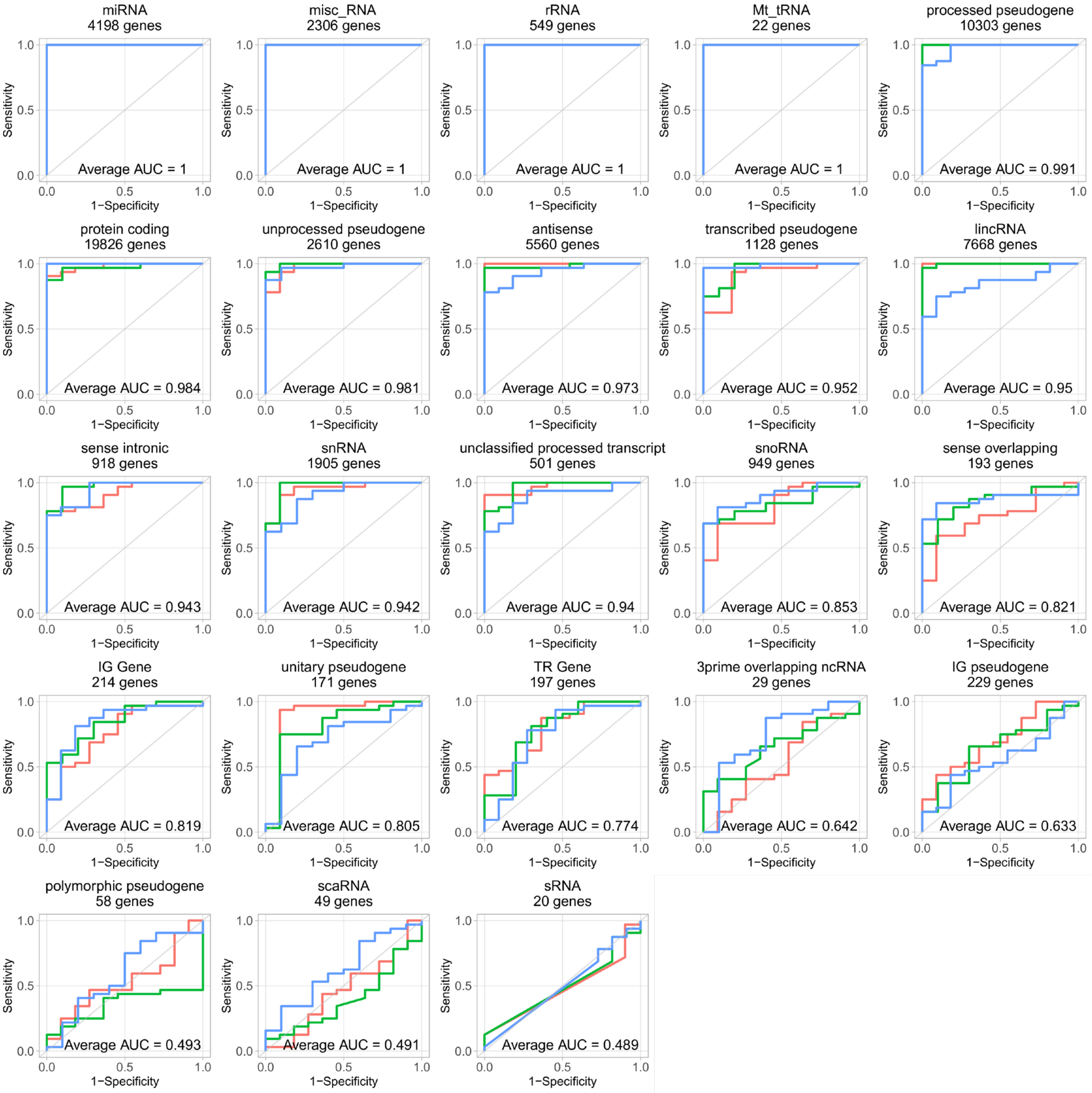

Fig. S14. Classification of cancer and normal serums by SVM. All the genes of each RNA type (panel title) were used as the features. Three ROC curves were generated from 3-fold cross validations.

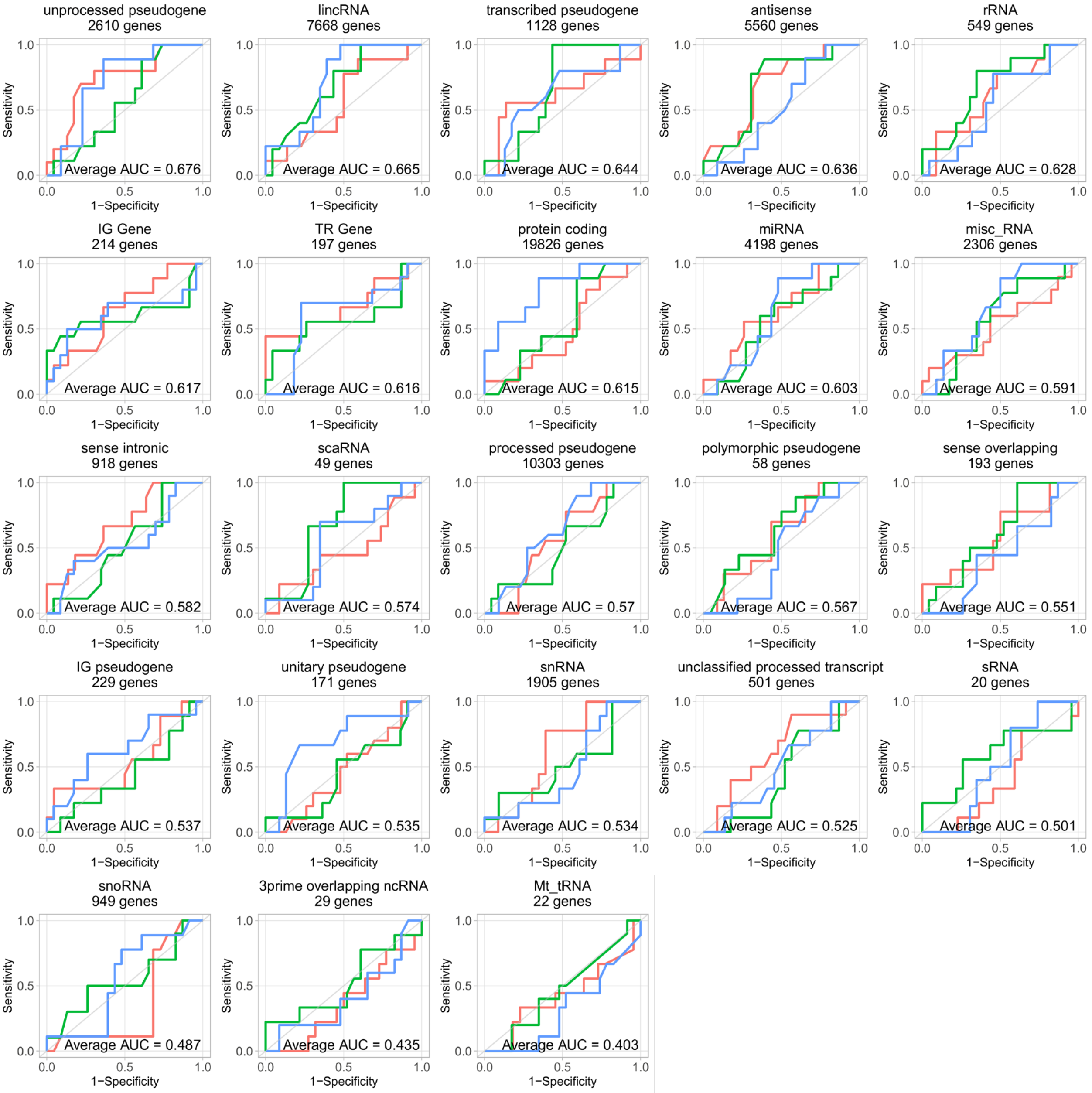

Fig. S15. Classification of recurrence and non-recurrence cancer samples by random forest. All the genes of each RNA type (panel title) were used as the features. Three ROC curves were generated from 3-fold cross validations.

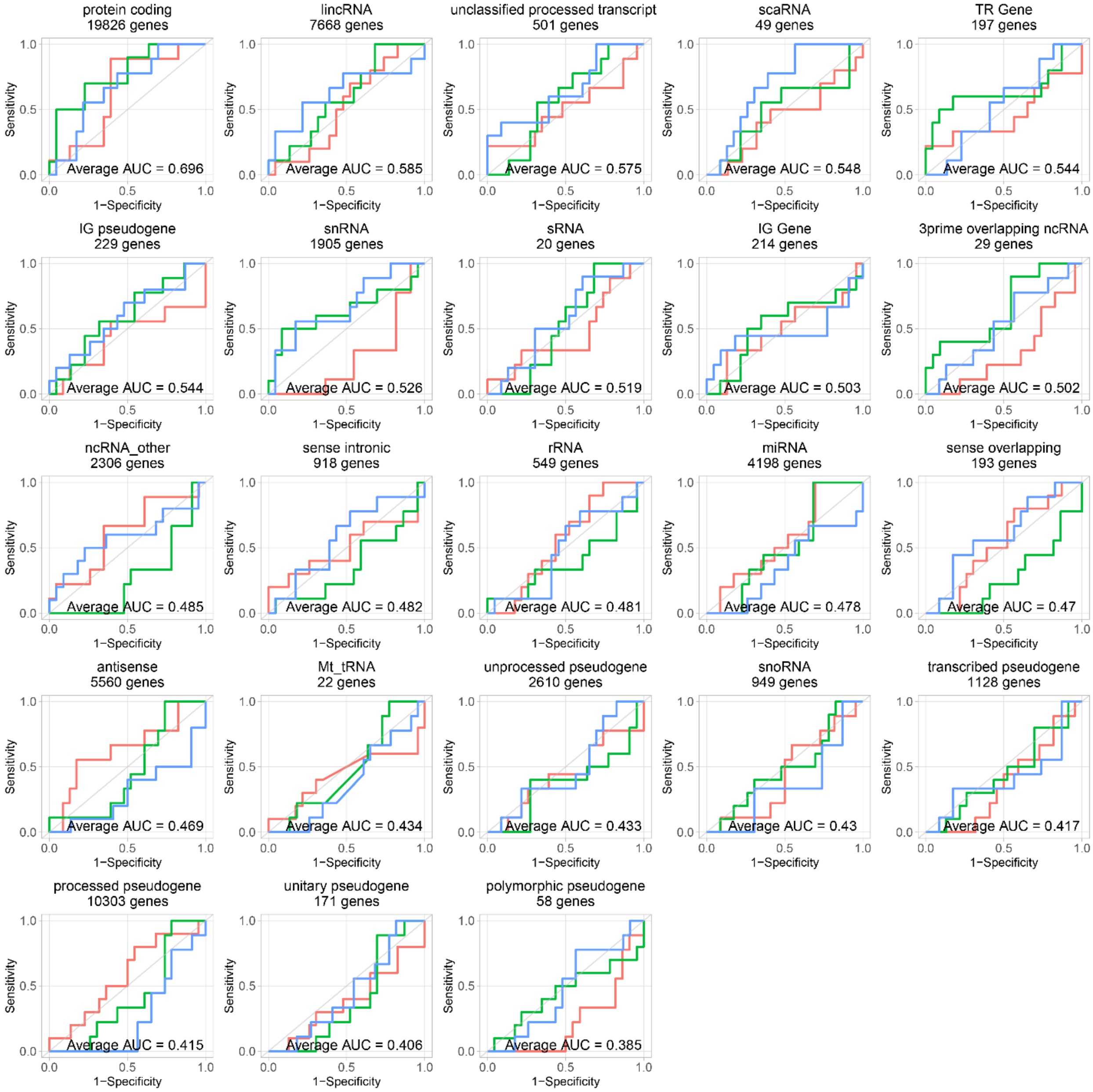

Fig. S16. Classification of recurrence and non-recurrence cancer samples by SVM. All the genes of each RNA type (panel title) were used as the features. Three ROC curves were generated from 3-fold cross validations.

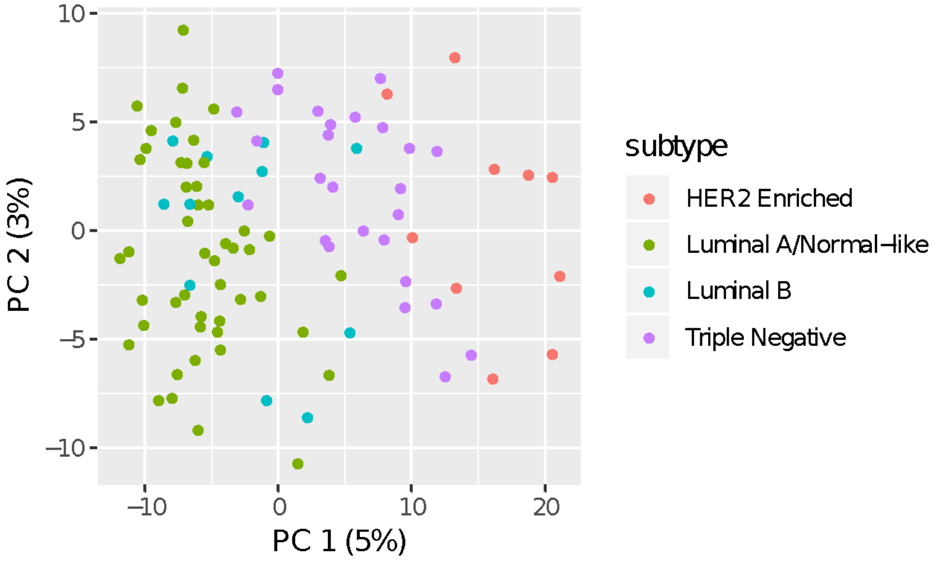

Fig. S17. Principle component analysis of the 96 samples (dots) based on 100 exRNAs. Her2-enriched, luminal A or normal-like, luminal B, and triple negative samples are colored coded in pink, green, blue, and purple, respectively.

Table S1. Contingency tables of exRNAs detected in RNA-seq-1, RNA-seq-2, and SILVER-seq. Corresponding to Figure S4B.

A

	SILVER-seq +	SILVER-seq -	Total
**RNA-seq-1 +**	1,706	673	2,379
**RNA-seq-1 -**	19,135	39,161	58,296
**Total**	20,841	39,834	60,675

Odds ratio = 5.188, Chi-squared p-value = 0

B

	SILVER-seq +	SILVER-seq -	Total
**RNA-seq-2 +**	2,933	1,567	4,500
**RNA-seq-2 -**	17,908	38,267	56,175
**Total**	20,841	39,834	60,675

Odds ratio = 4.000, Chi-squared p-value = 0

C

	SILVER-seq +	SILVER-seq -	Total
**RNA-seq-1 + OR RNA-seq-2 +**	4,200	2,116	6,316
**Other genes**	16,641	37,718	54,359
**Total**	20,841	39,834	60,675

Odds ratio = 4.499, Chi-squared p-value = 0

D

	SILVER-seq +	SILVER-seq -	Total
**RNA-seq-1 + AND RNA-seq-2 +**	439	124	563
**Other genes**	20,402	39,710	60,112
**Total**	20,841	39,834	60,675

Odds ratio =6.891, Chi-squared p-value = 2.572e-106

Table S2. Contingency tables of exRNAs detected in SILVER-seq-1, SILVER-seq-2, and RNA-seq-3. Corresponding to Figure S4D.

A

	RNA-seq-3 +	RNA-seq-3 -	Total
**SILVER-seq-1 +**	1,804	23,086	24,890
**SILVER-seq-1**	435	35,350	35,785
**Total**	2,239	58,436	60,675

Odds ratio = 6.350, Chi-squared p-value = 0

B

	RNA-seq-3 +	RNA-seq-3 -	Total
**SILVER-seq-2 +**	1,519	16,165	17,684
**SILVER-seq-2 -**	720	42,271	42,991
**Total**	2,239	58,436	60,675

Odds ratio = 5.517, Chi-squared p-value = 0

C

	RNA-seq-3 +	RNA-seq-3 -	Total
**SILVER-seq-1 + OR SILVER-seq-2 +**	1,992	28,116	30,108
**Other genes**	247	30,320	30,567
**Total**	2,239	58,436	60,675

Odds ratio = 8.697, Chi-squared p-value = 4.89e-315

D

	RNA-seq-3 +	RNA-seq-3 -	Total
**SILVER-seq-1 + AND SILVER-seq-2 +**	1,331	11,135	12,466
**Other genes**	908	47,301	48,209
**Total**	2,239	58,436	60,675

Odds ratio = 6.227, Chi-squared p-value = 0

Table S3. Summary of SILVER-seq data from 128 serum samples. C-R Status: Serum collected from the cancer patients who had cancer recurrences within 5 years of chemotherapy. C-N: Serum collected from the cancer patients who did not exhibit cancer recurrence during a 5-year follow-up after chemotherapy. N: serum from normal donors.

Sample ID	Status	Total number of reads	Number of uniquely mapped reads	Uniquely mapped reads %
**C1**	C-R	7,293,272	6,044,825	82.88%
**C2**	C-R	7,870,895	6,526,361	82.92%
**C3**	C-R	6,622,312	5,620,525	84.87%
**C4**	C-R	8,248,171	7,041,599	85.37%
**C5**	C-R	9,233,397	7,922,603	85.80%
**C6**	C-R	7,156,841	6,140,656	85.80%
**C7**	C-R	8,130,653	6,858,594	84.35%
**C8**	C-R	8,374,268	7,298,256	87.15%
**C9**	C-R	7,637,570	6,504,379	85.16%
**C10**	C-R	7,110,637	5,885,638	82.77%
**C11**	C-R	6,848,186	5,824,106	85.05%
**C12**	C-R	7,093,422	5,976,848	84.26%
**C13**	C-R	6,609,194	5,525,581	83.60%
**C14**	C-R	8,330,933	6,986,899	83.87%
**C15**	C-R	7,620,354	6,510,882	85.44%
**C16**	C-R	7,365,425	6,222,656	84.48%
**C17**	C-R	9,045,224	7,435,542	82.20%
**C18**	C-R	7,105,263	6,173,926	86.89%
**C19**	C-R	8,346,173	7,032,824	84.26%
**C20**	C-R	7,786,528	6,693,048	85.96%
**C21**	C-R	11,070,218	9,545,714	86.23%
**C22**	C-R	5,636,176	4,752,813	84.33%
**C23**	C-R	7,344,237	6,119,400	83.32%
**C24**	C-R	7,389,168	6,166,270	83.45%
**C25**	C-R	7,023,051	5,955,431	84.80%
**C26**	C-R	7,799,765	6,661,309	85.40%
**C27**	C-R	8,827,999	7,634,037	86.48%
**C28**	C-R	8,161,797	6,876,973	84.26%
**C29**	C-N	7,646,966	5,784,958	75.65%
**C30**	C-N	7,382,725	6,330,528	85.75%
**C31**	C-N	8,429,401	7,308,056	86.70%
**C32**	C-N	7,649,365	6,471,495	84.60%
**C33**	C-N	6,335,158	5,350,491	84.46%

**C34**	C-N	6,891,717	5,709,138	82.84%
**C35**	C-N	8,340,748	7,187,823	86.18%
**C36**	C-N	7,315,306	6,017,481	82.26%
**C37**	C-N	8,203,775	6,991,452	85.22%
**C38**	C-N	8,505,871	7,105,154	83.53%
**C39**	C-N	8,491,263	7,259,373	85.49%
**C40**	C-N	7,630,757	6,124,622	80.26%
**C41**	C-N	8,724,735	7,454,713	85.44%
**C42**	C-N	7,672,233	6,663,618	86.85%
**C43**	C-N	8,401,167	7,147,021	85.07%
**C44**	C-N	7,116,876	6,046,207	84.96%
**C45**	C-N	5,791,185	4,802,272	82.92%
**C46**	C-N	6,715,321	5,721,536	85.20%
**C47**	C-N	9,147,154	7,901,213	86.38%
**C48**	C-N	7,820,560	6,747,814	86.28%
**C49**	C-N	8,047,948	6,857,495	85.21%
**C50**	C-N	7,215,702	6,066,533	84.07%
**C51**	C-N	8,048,885	6,949,432	86.34%
**C52**	C-N	6,955,775	5,502,735	79.11%
**C53**	C-N	7,002,006	5,271,164	75.28%
**C54**	C-N	6,274,345	5,195,457	82.80%
**C55**	C-N	9,586,903	8,247,657	86.03%
**C56**	C-N	8,328,434	7,122,153	85.52%
**C57**	C-N	6,820,883	5,725,572	83.94%
**C58**	C-N	8,798,580	7,656,525	87.02%
**C59**	C-N	7,772,216	6,245,079	80.35%
**C60**	C-N	6,514,249	5,296,154	81.30%
**C61**	C-N	7,857,570	6,793,867	86.46%
**C62**	C-N	7,322,319	6,158,949	84.11%
**C63**	C-N	7,570,788	6,382,468	84.30%
**C64**	C-N	7,458,148	6,442,717	86.38%
**C65**	C-N	10,165,886	8,612,608	84.72%
**C66**	C-N	7,355,997	6,413,125	87.18%
**C67**	C-N	8,561,951	7,258,003	84.77%
**C68**	C-N	6,459,683	5,333,914	82.57%
**C69**	C-N	6,047,318	4,679,201	77.38%
**C70**	C-N	6,628,185	5,665,939	85.48%
**C71**	C-N	7,935,080	6,688,680	84.29%
**C72**	C-N	6,392,908	5,296,188	82.84%

**C73**	C-N	7,846,486	6,495,130	82.78%
**C74**	C-N	7,677,529	6,546,753	85.27%
**C75**	C-N	7,578,732	6,479,616	85.50%
**C76**	C-N	9,298,701	7,706,716	82.88%
**C77**	C-N	7,986,157	6,793,437	85.07%
**C78**	C-N	5,873,688	4,867,513	82.87%
**C79**	C-N	7,804,645	6,651,239	85.22%
**C80**	C-N	6,103,776	4,900,734	80.29%
**C81**	C-N	6,968,233	5,516,174	79.16%
**C82**	C-N	7,802,809	6,445,292	82.60%
**C83**	C-N	7,288,621	5,502,550	75.50%
**C84**	C-N	7,764,460	6,500,442	83.72%
**C85**	C-N	7,283,528	6,229,155	85.52%
**C86**	C-N	8,761,199	7,287,353	83.18%
**C87**	C-N	8,296,427	7,121,670	85.84%
**C88**	C-N	6,965,863	5,885,477	84.49%
**C89**	C-N	6,536,128	5,514,069	84.36%
**C90**	C-N	6,532,672	4,995,183	76.46%
**C91**	C-N	7,377,949	6,212,975	84.21%
**C92**	C-N	6,490,139	5,229,779	80.58%
**C93**	C-N	9,183,238	7,910,546	86.14%
**C94**	C-N	8,961,017	7,675,137	85.65%
**C95**	C-N	9,025,676	7,600,017	84.20%
**C96**	C-N	8,148,798	6,940,255	85.17%
**N1**	N	9,263,850	7,883,088	85.10%
**N2**	N	8,862,600	7,309,574	82.48%
**N3**	N	7,524,176	5,704,954	75.82%
**N4**	N	8,087,304	6,787,790	83.93%
**N5**	N	9,212,857	7,916,254	85.93%
**N6**	N	9,459,977	8,084,035	85.46%
**N7**	N	8,873,306	6,506,505	73.33%
**N8**	N	9,647,151	7,914,755	82.04%
**N9**	N	10,720,753	8,372,183	78.09%
**N10**	N	8,960,995	7,402,048	82.60%
**N11**	N	10,753,306	7,408,864	68.90%
**N12**	N	6,838,430	4,927,597	72.06%
**N13**	N	8,016,497	5,224,664	65.17%
**N14**	N	7,248,737	5,116,580	70.59%
**N15**	N	9,317,317	7,675,978	82.38%

**N16**	N	8,843,226	6,532,444	73.87%
**N17**	N	8,434,973	6,172,272	73.17%
**N18**	N	9,288,594	7,687,495	82.76%
**N19**	N	8,630,942	6,698,816	77.61%
**N20**	N	9,595,997	7,987,941	83.24%
**N21**	N	6,312,539	5,105,886	80.88%
**N22**	N	7,039,294	5,621,452	79.86%
**N23**	N	8,815,317	7,204,811	81.73%
**N24**	N	7,331,502	5,925,628	80.82%
**N25**	N	9,740,427	8,104,113	83.20%
**N26**	N	7,231,993	5,758,838	79.63%
**N27**	N	10,230,936	8,327,144	81.39%
**N28**	N	8,579,794	7,286,744	84.93%
**N29**	N	10,808,607	8,789,442	81.32%
**N30**	N	7,312,205	5,604,035	76.64%
**N31**	N	9,232,461	6,992,056	75.73%
**N32**	N	8,964,442	7,278,097	81.19%

Table S4. Summary of the 96 breast cancer serum samples. Recurrence status R: Serum collected from the cancer patients who had cancer recurrences within 5 years of chemotherapy. Recurrence Status N: Serum collected from the cancer patients who did not exhibit cancer recurrence during a 5-year follow-up after chemotherapy. Chemo-, GnRH (Gonadotropin-releasing hormone), TAM (Tamoxifen), AI (Aromatase inhibitors) and BSO (Bilateral salpingo-oophorectomy) therapy status B: Serum was collected before the treatment started. Therapy status D: Serum was collected while the treatment was in progress. Therapy status A: Serum was collected after the treatment ended. Therapy status N/A: No record or not applicable.

Sample ID	Recurrence status	Cancer subtype	Chemo-therapy status	GnRH therapy status	TAM therapy status	AI therapy status	BSO therapy status
**C1**	R	Triple Negative	D	N/A	N/A	N/A	N/A
**C2**	R	Triple Negative	A	N/A	N/A	N/A	N/A
**C3**	R	Triple Negative	A	N/A	N/A	N/A	N/A
**C4**	R	Triple Negative	A	N/A	N/A	N/A	N/A
**C5**	R	Luminal A/Normal-like	D	B	B	N/A	N/A
**C6**	R	Luminal A/Normal-like	A	B	N/A	N/A	N/A
**C7**	R	Luminal A/Normal-like	A	N/A	N/A	N/A	N/A
**C8**	R	Luminal A/Normal-like	A	N/A	B	N/A	N/A
**C9**	R	Luminal A/Normal-like	A	N/A	N/A	N/A	N/A
**C10**	R	Luminal A/Normal-like	A	N/A	N/A	N/A	N/A
**C11**	R	Luminal A/Normal-like	A	N/A	N/A	N/A	N/A
**C12**	R	Triple Negative	D	N/A	N/A	N/A	N/A
**C13**	R	Triple Negative	A	N/A	N/A	N/A	N/A
**C14**	R	Triple Negative	A	N/A	N/A	N/A	N/A
**C15**	R	Triple Negative	A	N/A	N/A	N/A	N/A
**C16**	R	Triple Negative	A	N/A	N/A	N/A	N/A
**C17**	R	Triple Negative	D	N/A	N/A	N/A	N/A
**C18**	R	Triple Negative	A	N/A	N/A	N/A	N/A
**C19**	R	Triple Negative	A	N/A	N/A	N/A	N/A
**C20**	R	Luminal A/Normal-like	A	N/A	B	N/A	N/A
**C21**	R	Luminal A/Normal-like	A	N/A	N/A	N/A	N/A
**C22**	R	HER2 Enriched	A	A	N/A	N/A	N/A
**C23**	R	Luminal A/Normal-like	A	B	N/A	N/A	N/A
**C24**	R	HER2 Enriched	A	A	N/A	N/A	N/A
**C25**	R	HER2 Enriched	D	D	N/A	N/A	N/A
**C26**	R	HER2 Enriched	A	A	N/A	N/A	N/A
**C27**	R	HER2 Enriched	A	A	N/A	N/A	N/A
**C28**	R	HER2 Enriched	A	A	N/A	N/A	N/A
**C29**	N	Luminal B	A	N/A	B	N/A	N/A

**C30**	N	Luminal B	A	N/A	N/A	N/A	N/A
**C31**	N	Luminal A/Normal-like	A	N/A	N/A	N/A	N/A
**C32**	N	Luminal A/Normal-like	A	N/A	N/A	N/A	N/A
**C33**	N	Luminal A/Normal-like	D	N/A	B	N/A	N/A
**C34**	N	Luminal A/Normal-like	A	N/A	N/A	N/A	N/A
**C35**	N	Luminal A/Normal-like	A	N/A	N/A	N/A	N/A
**C36**	N	Luminal A/Normal-like	B	B	N/A	N/A	N/A
**C37**	N	Triple Negative	A	N/A	N/A	N/A	B
**C38**	N	Triple Negative	A	N/A	N/A	N/A	B
**C39**	N	Luminal B	A	N/A	B	N/A	N/A
**C40**	N	Luminal B	A	N/A	N/A	N/A	N/A
**C41**	N	Triple Negative	A	D	N/A	N/A	N/A
**C42**	N	Triple Negative	A	A	N/A	N/A	N/A
**C43**	N	Luminal A/Normal-like	D	N/A	N/A	N/A	B
**C44**	N	Luminal A/Normal-like	A	N/A	N/A	N/A	B
**C45**	N	Luminal A/Normal-like	D	N/A	B	N/A	N/A
**C46**	N	Luminal A/Normal-like	A	N/A	N/A	N/A	N/A
**C47**	N	Luminal A/Normal-like	D	N/A	N/A	N/A	N/A
**C48**	N	Luminal A/Normal-like	A	N/A	N/A	N/A	N/A
**C49**	N	Triple Negative	D	N/A	N/A	N/A	N/A
**C50**	N	Triple Negative	A	N/A	N/A	N/A	N/A
**C51**	N	Triple Negative	A	N/A	N/A	N/A	N/A
**C52**	N	Triple Negative	A	N/A	N/A	N/A	N/A
**C53**	N	Luminal B	D	N/A	B	N/A	N/A
**C54**	N	Luminal B	A	N/A	N/A	N/A	N/A
**C55**	N	Luminal A/Normal-like	D	D	B	N/A	N/A
**C56**	N	Luminal A/Normal-like	A	A	B	N/A	N/A
**C57**	N	Luminal A/Normal-like	D	N/A	N/A	N/A	B
**C58**	N	Luminal A/Normal-like	A	N/A	N/A	N/A	B
**C59**	N	Luminal A/Normal-like	D	N/A	B	N/A	N/A
**C60**	N	Luminal A/Normal-like	A	N/A	N/A	N/A	N/A
**C61**	N	Luminal B	D	N/A	B	N/A	N/A
**C62**	N	Luminal B	A	N/A	B	N/A	N/A
**C63**	N	Luminal A/Normal-like	A	B	N/A	N/A	N/A
**C64**	N	Luminal A/Normal-like	A	N/A	N/A	N/A	N/A
**C65**	N	Luminal A/Normal-like	A	B	N/A	B	N/A
**C66**	N	Luminal A/Normal-like	A	N/A	N/A	N/A	N/A
**C67**	N	HER2 Enriched	A	N/A	N/A	N/A	N/A
**C68**	N	HER2 Enriched	A	N/A	N/A	N/A	N/A

**C69**	N	Luminal B	D	N/A	B	N/A	N/A
**C70**	N	Luminal B	A	N/A	B	N/A	N/A
**C71**	N	HER2 Enriched	D	N/A	N/A	N/A	N/A
**C72**	N	HER2 Enriched	A	N/A	N/A	N/A	N/A
**C73**	N	Luminal A/Normal-like	D	N/A	B	N/A	N/A
**C74**	N	Luminal A/Normal-like	A	N/A	N/A	N/A	N/A
**C75**	N	Triple Negative	D	N/A	N/A	N/A	N/A
**C76**	N	Triple Negative	A	N/A	N/A	N/A	N/A
**C77**	N	Luminal A/Normal-like	A	N/A	B	N/A	N/A
**C78**	N	Luminal A/Normal-like	A	N/A	N/A	N/A	N/A
**C79**	N	Triple Negative	D	N/A	N/A	N/A	N/A
**C80**	N	Triple Negative	A	N/A	N/A	N/A	N/A
**C81**	N	Luminal A/Normal-like	A	B	D	N/A	N/A
**C82**	N	Luminal A/Normal-like	A	B	D	N/A	N/A
**C83**	N	Luminal A/Normal-like	D	N/A	B	N/A	N/A
**C84**	N	Luminal A/Normal-like	A	N/A	N/A	N/A	N/A
**C85**	N	Luminal A/Normal-like	D	N/A	B	N/A	N/A
**C86**	N	Luminal A/Normal-like	A	N/A	N/A	N/A	N/A
**C87**	N	Triple Negative	D	N/A	N/A	N/A	N/A
**C88**	N	Triple Negative	A	N/A	N/A	N/A	N/A
**C89**	N	Luminal A/Normal-like	A	N/A	N/A	N/A	N/A
**C90**	N	Luminal A/Normal-like	A	N/A	N/A	N/A	N/A
**C91**	N	Luminal A/Normal-like	A	N/A	B	N/A	N/A
**C92**	N	Luminal A/Normal-like	A	N/A	N/A	N/A	N/A
**C93**	N	Luminal A/Normal-like	D	N/A	B	N/A	N/A
**C94**	N	Luminal A/Normal-like	A	N/A	B	N/A	N/A
**C95**	N	Luminal B	D	N/A	B	N/A	N/A
**C96**	N	Luminal B	A	N/A	B	N/A	N/A

Table S5. Prior-association genes. The genes that have been associated with breast cancer by prior literature.

Category	Gene name
**H/I™ [1]**	*HOXB13*, *IL17BR*
**PAM50 [2]**	*UBE2T*, *BIRC5*, *NUF2*, *CDC6*, *CCNB1*, *TYMS*, *MYBL2*, *CEP55*, *MELK*, *NDC80*, *RRM2*, *UBE2C*, *CENPF*, *PTTG1*, *EXO1*, *ORC6L*, *ANLN*, *CCNE1*, *CDC20*, *MKI67*, *KIF2C*, *ACTR3B*, *MYC*, *EGFR*, *KRT5*, *PHGDH*, *CDH3*, *MIA*, *KRT17*, *FOXC1*, *SFRP1*, *KRT14*, *ESR1*, *SLC39A6*, *BAG1*, *MAPT*, *PGR*, *CXXC5*, *MLPH*, *BCL2*, *MDM2*, *NAT1*, *FOXA1*, *BLVRA*, *MMP11*, *GPR160*, *FGFR4*, *GRB7*, *TMEM45B*, *ERBB2*;
**Onco*type* DX™ [3]**	*BAG1*, *BCL2*, *CCNB1*, *CD68*, *SCUBE2*, *CTSL2*, *ESR1*, *GRB7*, *GSTM1*, *ERBB2*,<br>*MKI67*, *MYBL2*, *PGR*, *AURKA*, *MMP11*, *BIRC5*, *ACTB*, *GAPDH*, *GUSB*, *RPLP0*, *TFRC*;
**BreastOncPx™ [4]**	*BUB1*, *CCNB1*, *CENPA*, *DC13*, *DIAPH3*, *MELK*, *MYBL2*, *ORC6L*, *PKMYT1*, *PRR11*, *RACGAP1*, *RFC4*, *TK1*, *UBE2S*;
**MammaPrint™ [5]**	*BBC3*, *EGLN1*, *TGFB3*, *ESM1*, *IGFBP5*, *FGF18*, *SCUBE2*, *TGFB3*, *WISP1*, *FLT1*, *HRASLS*, *STK32B*, *RASSF7*, *DCK*, *MELK*, *EXT1*, *GNAZ*, *EBF4*, *MTDH*, *PITRM1*, *QSCN6L1*, *CCNE2*, *ECT2*, *CENPA*, *LIN9*, *KNTC2*, *MCM6*, *NUSAP1*, *ORC6L*, *TSPYL5*, *RUNDC1*, *PRC1*, *RFC4*, *RECQL5*, *CDCA7*, *DTL*, *COL4A2*, *GPR180*, *MMP9*, *GPR126*, *RTN4RL1*, *DIAPH3*, *CDC42BPA*, *PALM2*, *ALDH4A1*, *LPCAT1*, *LPCAT2*, *LPCAT4*, *OXCT1*, *PECI*, *GMPS*, *GSTM3*, *SLC2A3*, *FLT1*, *FGF18*, *COL4A2*, *GPR180*, *EGLN1*, *MMP9*, *C9orf30*, *ZNF533*, *C16orf61*, *SERF1A*, *C20orf46*, *LGP2*, *NMU*, *UCHL5*, *JHDM1D*, *AP2B1*, *MS4A7*, *RAB6B*;
**HDPP [6]**	*ABCD4*, *ADD3*, *AFP*, *AFTIPHILIN*, *ALAS2*, *ANPEP*, *ARHGAP19*, *ARIH2*, *ATOH8*, *ATP8B2*, *AXIN2*, *BIN3*, *BMP4*, *C11orf57*, *C12orf29*, *C14orf49*, *C17orf68*, *C6orf188*, *C6orf190*, *C7orf25*, *CASP6*, *CD3D*, *CD55*, *CD69*, *CDON*, *CFL2*, *CLCN7*, *CLSTN3*, *COL8A1*, *COLEC12*, *COMP*, *CRMP1*, *CX3CR1*, *CXCR4*, *CYorf15B*, *DCBLD1*, *DDEF2*, *DKFZp686E10196*, *DMN*, *DPYSL2*, *DRCTNNB1A*, *EDG5*, *EDNRB*, *ELMO3*, *EPB41*, *ETS1*, *FAIM3*, *FAM46A*, *FAM89A*, *FGF2*, *FLNC*, *FYN*, *GAS7*, *GLRX2*, *GLTSCR1*, *GPC3*, *GPR172A*, *GPX3*, *GRB7*, *GRHL1*, *GYPC*, *HEMGN*, *HHIP*, *HMHA1*, *HSPBP1*, *IL17RD*, *INHBA*, *IRF6*, *ITGA4*, *ITM2A*, *KIAA1549*, *KIAA1754L*, *KIAA1826*, *KIRREL*, *KLF9*, *KPNA3*, *LAD1*, *LAT*, *LOC116143*, *LOC133308*, *LOC90624*, *LONRF1*, *LPGAT1*, *LTBP1*, *LTBP4*, *MAP4*, *MAP7D3*, *MARCH8*, *MED19*, *MFAP4*, *MGC40579*, *MLXIPL*, *MTHFR*, *NDN*, *NLGN2*, *NUDT9*, *OSBPL3*, *PDE2A*, *PELI2*, *PERLD1*, *PEX11B*, *PGM2L1*, *PIM1*, *PKP3*, *PLAC8*, *PLAU*, *PLXNA4B*, *PPP1R14A*, *PPP5C*, *PRKCA*, *PRSS12*, *PTPRCAP*, *PXDN*, *RAC2*, *RASIP1*, *RBM5*, *RBP4*, *RFT1*, *RNF166*, *S100A11*, *S100B*, *SDPR*, *SEMA6A*, *SEMA7A*, *SENP5*, *SFRS5*, *SH3BP5*, *SH3KBP1*, *SH3YL1*, *SLC16A7*, *SLC25A42*, *SLC38A5*, *SLK*, *SNCA*, *SNF1LK2*, *SOCS1*, *SOX10*, *SPTBN2*, *ST3GAL2*, *ST6GALNAC6*, *STARD4*, *STAT5A*, *STON2*, *STXBP1*, *TCF7*, *TF*, *TGFBR3*, *TIGA1*, *TLE4*, *TMEFF1*, *TSPAN7*, *UBE2G1*, *WDHD1*, *ZAP70*, *ZNF276*, *ZNF281*, *ZNF609*, *ZNF690*;

**HTICS** [7]	*AURKB*, *CCNA2*, *SCRN1*, *NPY*, *ATP7B*, *CHAF1B*, *CCNB1*, *CLDN8*, *NRP1*, *CCR2*, *C1QB*, *CD74*, *VCAM1*, *CD180*, *ITGB2*, *CD72*, *ST8SIA4*;
**eXagen*BC*™** [8]	*CYP2D6*, *CYP24*, *PDCD6IP*, *BIRC5*, *NR1D1*, *SMARCE1*;
**Mammostrat™** [9]	*TP53*, *NDRG1*, *CEACAM5*, *SLC7A2*, *SLC7A5*, *TRMT2A*;
**ProEx™Br** [10]	*E2F1*, *RASA1*, *PSMB1*;
**Rotterdam** [11]	*CLN8*, *GOLM1*, *YIF1A*, *IL18*, *CBX3*, *DUSP4*, *PPP1CC*, *ABLIM1*, *TNFSF13*, *TMEM8A*, *C3*, *NCAPG2*, *C11orf9*, *MAP4*, *TACC2*, *SMC4*, *OR12D2*, *ORC3*, *ATAD2*, *SMC4*, *DUSP4*, *CD44*, *PLK1*, *CNKSR1*, *Hs.591790*, *FEN1*, *FKBP2*, *KPNA2*, *RRNAD1*, *GFOD2*, *AP2A2*, *MLF1IP*, *NEURL*, *SUPT16H*, *ZFP36L2*, *ARHGDIB*, *PSMC2*, *ZCCHC8*, *ANKHD1-EIF4EBP3*, *CD44*, *SLC35A1*, *CCNE2*, *PHF11*, *POLQ*, *CAPN2*, *ETV2*, *UCKL1*, *LST1*, *ACACB*, *Hs.505202*, *EEF1A2*, *NEFL*, *TNFSF13*, *MMP23B*, *GTSE1*, *RFX7*, *COL2A1*, *GAS2*, *ZNF362*, *FUT3*, *MYH2*, *TNFSF10*, *RPL23AP7*, *GABRQ*, *PARP4*, *C11orf51*, *BCL2L14*, *CEP57*, *RPS4XP3*, *ACOT11*;
**MapQuant Dx™** [12]	*UBE2C*, *KPNA2*, *TPX2*, *FOXM1*, *STK6*, *CCNA2*, *BIRC5*, *MYBL2*;
**IGS™** [13]	*DPF2*, *CASP8*, *BCL2*, *SCGN*, *SWAP70*, *KIAA0276*, *C10orf9*, *C10orf7*, *TOB2*, *XPR1*, *CD59*, *LRP2*, *PLP2*, *MAPK14*, *CXCL2*, *MMP7*, *MGP*, *MLF1*, *FLNB*, *SCNM1*, *HSPC163*, *C5orf18*, *MGC4399*, *CDW92*, *TMC4*, *ZDHHC2*, *TICAM2*, *KDELR3*, *GNPDA1*, *THEM2*, *DBR1*, *FLJ90709*, *FLJ10774*, *C16orf33*, *GAPD*, *LDHA*, *MR1*, *LARS*, *GTPBP1*, *PRSS16*, *WFDC2*, *AIM1*, *DHRS6*, *DHRS4*, *MGC15429*, *MGC45840*, *ECHDC2*, *GOLGA8B*, *GOLGA8A*, *KIAA0436*, *CYP4V2*, *JTV1*, *ICMT*, *DNMT3A*, *HNMT*, *METTL7A*, *METTL2*, *VIL2*, *TPD52*, *ARPC5*, *NOL8*, *NSF*, *RAD23B*, *SRP54*, *HSPA2*, *THAP2*, *CIRBP*, *SNRPN*, *KIAA0052*, *DUSP10*, *SSR1*, *ERBB4*, *EMP1*, *CHPT1*, *LRPAP1*, *FLJ11752*, *CSTF1*, *KLHL20*, *DNAJC13*, *APLP2*, *ARGBP2*, *DNAJB1*, *NEBL*, *SH3BGRL*, *NUDT5*, *GABARAPL1*, *MAPT*, *DCBLD1*, *STK39*, *PAK2*, *CSNK2A1*, *PILRB*, *ERN1*, *SGK3*, *WEE1*, *MAST4*, *C11orf17*, *NUP37*, *CLTC*, *COPB2*, *SLC25A25*, *ECOP*, *PDE8A*, *STAM*, *TUBB*, *SNX6*, *RAB23*, *PLAA*, *STC2*, *LTF*, *ISGF3G*, *ATXN3*, *GTF3C3*, *GSK3B*, *KLF10*, *ELL2*, *ZBTB20*, *IRX3*, *ETS1*, *SERTAD1*, *MGC4251*, *MAFF*, *SFPQ*, *CITED4*, *CEBPD*, *EIF4E2*, *HS2ST1*, *AGPS*, *PGK1*, *ATIC*, *ETNK1*, *ALG2*, *NCE2*, 8-*Mar*, *CNOT4*, *RNF8*, *PSMA5*, *DPF2*, *AMMECR1*, *KIAA1287*, *LOC144233*, *NPAS*-4, *FLJ20530*, *THUMPD3*, *MGC45564*, *CAP350*, *ETAA16*, *HAN11*, *DNAPTP6*, *C7orf25*, *FLJ37953*, *FLJ10587*, *C7orf36*, *ELP4*, *NDEL1*, *NPD014*, *DKFZP564D172*, *FAM53C*, *IER5*, *LOC255783*, *KIAA0146*, *KIAA0792*, *LOC283481*, *CG018*, *LOC130576*, *NGFRAP1L1*, *KIAA1217*, *C4orf7*, *C21orf86*, *C9orf64*, *FLJ13456*, *KIAA1600*, B7-H4, *C7orf2*, *NUCKS*, *DKFZP566D1346*, *LOC388279*, *FLJ31795*, *C6orf107*, *FLJ12439*, *FLJ12806*, *FLJ39370*;
**NuvoSelect™** [14]	*DD96*, *FCGR3A*, *PLD3*, *CCNL2*, *PP591*, *SEC61A1*, *KPNA2*, *NFAT5*, *HN1*, *DKFZP564I1171*, *SLC35B1*, *PCDHB2*, *NME1*, *EFEMP1*, *SLC35B1*, *RALGDS*, *SURF4*, *KRT13*, *FLJ13855*, *GSR*, *ATF5*, *LCHN*, *PTPRC*, *ACLY*, *FAAH*, *RTKN*, *FLJ20323*, *STOML1*, *MGST1*, *CREM*, *PB1*, *ANXA8*, *FLJ11196*, *HSPC134*, *HBOA*, *TFRC*, *TCEB3*, *APH1A*, *KIAA1041*, *PRPSAP1*, *EPB42*, *KIAA0742*, *NME2*, *TBPL1*, *GTF2H3*, *SLC7A10*, *NDP*, *SCARA3*, *APOE*, *DKFZP434F195*, *KIAA1155*;

**Protein expression associated with breast cancer**	*TOP2A*, *EGFR*; [15]<br>*FOXA1*; [16]<br>*PIP*; [17]<br>*BigH3*; [18]<br>*MCM2*; [19]<br>*HERV-K*; [20]<br>*KI67*; [21]<br>*CAP2*; [22]<br>*SNCG*; [23]<br>*DAP*; [24]<br>*CENP-A*; [25]<br>*MDC1*; [26]<br>*PRKCA*; [27]<br>*YB-1*; [28]<br>*5-LOX*; [29]<br>*GRB7*, *HER2*; [30]<br>*MKI67*, *CCNA1*, *CCNA2*, *CCNB1*, *CCNB2*, *CCNB3*, *CCNC*, *CCND1*, *CCND2*, *CCND3*, *CCNE1*, *CCNE2*, *CCNF*, *CCNG1*, *CCNG2*, *CCNH*, *CCNI*, *CCNI2*, *CCNJ*, *CCNJL*, *CCNK*, *CCNL1*, *CCNL2*, *CCNO*, *CCNT1*, *CCNT2*, *CCNY*, *CCNYL1*, *CCNYL2*, *CCNYL3*, *PLAU*, *TP53*, *CDKN1A*, *CASP9*, *BAK1*, *BAD*, *BCL2L11*, *BCL2A1*, *BCL2L1*, *CASP4*, *AIFM1*, *TIAF1*, *BCL2*, *CASP3*, *SIVA1*, *BAX*, *PAWR*, *BRCA1*, *BRCA2*; [31]<br>*ESR1*, *ESR2*, *PGR*, *AR*; [32]<br>*NME1*; [33]
**RNA expression associated with breast cancer**	*PTPN13*, *PTPL1*; [34]<br>*EPS15*; [35] *WDR5*; [36]<br>*PVRL4*; [37]<br>*DLC1*, *CDK6*; [38]<br>*INPP4B*; [39]<br>*CCNE1*; [40]<br>*LINC00472*; [41]<br>*EPB41L-AS2*; [42]<br>*MEG3*; [43]<br>*CCAT1*; [44]<br>*BCYRN1*; [45]<br>*FGF14-AS2*; [46]<br>*H19*; [47]<br>*CCAT2*; [48]<br>*GAS5*; [49]<br>*BCAR4*; [50]<br>*MALAT1*; [51]<br>*BTG3*; [52]<br>*ABCB10*; [53]<br>*LINC-ROR*; [54]<br>*TWIST2*; [55]<br>*TWISTNB*; [56]

**DNA sequence associated with breast cancer**	*AATK*, *USP32*, *ACTB*, *C20orf112*, *APOL1*, *CD74*, *MBD6*, *GNB2*, *CTSD*, *KRT18*, *PLEC*, *TSPAN14*, *YWHAZ*, *HLA-E*, *ZBTB33*, *COL1A1*, *FMNL3*, *CYB5R3*, TXNIP, *DCLK1*, *COL3A1*, *ELF3*, *SLC39A6*, *MGP*, *NCRNA00188*, *STC2*, *RNF11*, *TAX1BP1*, *MALAT1*; [57]<br>*ACHE*, *EPHB4*, *BCHE*, *MME*; [58]<br>*TOP2A*; [15]<br>*ADPRHL1*, *DCUN1D2*, *TMCO3*, *TFDP1*; [59]<br>*TP53*; [60]<br>*BRCA1*, *APC*, *RASSF1A*, *WIF1*, *MGMT*, *MAL*, *CDH13*, *RARβ*, *CDKN2A*, *TP73*, *GSTP1*, *CDH1*; [61]<br>*CCND2*, *RARB*, *TWIST1*; [62]<br>*CYP1B1*, *ARHI*; [63]<br>*PIK3CA*; [64]<br>*BRCA2*; [65]<br>*PTEN*; [66]<br>*RAD50*; [67]<br>*CHEK2*, *PALB2*; [68]<br>*RAD51C*; [69]<br>*NBN*; [70]<br>*ATM*; [71]<br>*FANCM*; [72]<br>*FGFR2*, *TOX3*, *MAP3K1*, *LSP1*, *HCN1*; [73]<br>*BRIP1*; [74]<br>*SULT1A1*; [75]<br>*RB1*; [76]<br>*VEGF*; [77]<br>*MDM2*; [78]<br>*HLA-G*; [79]<br>*IL1A*; [80]<br>*PDCD6IP*; [81]<br>*RAD51*; [82]<br>*APOBEC3A*, *APOBEC3AP1*, *APOBEC3B*, *APOBEC3B-AS1*, *APOBEC3C*, *APOBEC3D*, *APOBEC3F*, *APOBEC3G*, *APOBEC3H*; [83]<br>*KRAS*, *NRAS*; [84]

References

1. Ma, X.J. et al. A two-gene expression ratio predicts clinical outcome in breast cancer patients treated with tamoxifen. *Cancer Cell* **5**, 607-616 (2004).
2. Wallden, B. et al. Development and verification of the PAM50-based Prosigna breast cancer gene signature assay. *BMC medical genomics* **8**, 54 (2015).
3. Rath, M.G. et al. Oncotype DX((R)) in breast cancer patients: clinical experience, outcome and follow-up-a case-control study. *Archives of gynecology and obstetrics* **297**, 443-447 (2018).
4. Garber, K. Genomic medicine. Gene expression tests foretell breast cancer's future. *Science* **303**, 1754-1755 (2004).
5. van de Vijver, M.J. et al. A gene-expression signature as a predictor of survival in breast cancer. *The New England journal of medicine* **347**, 1999-2009 (2002).
6. Staaf, J. et al. Identification of subtypes in human epidermal growth factor receptor 2--positive breast cancer reveals a gene signature prognostic of outcome. *Journal of clinical oncology : official journal of the American Society of Clinical Oncology* **28**, 1813-1820 (2010).
7. Liu, J.C. et al. Seventeen-gene signature from enriched Her2/Neu mammary tumor-initiating cells predicts clinical outcome for human HER2+:ERalpha- breast cancer. *Proc Natl Acad Sci U S A* **109**, 5832-5837 (2012).
8. Davis, L.M. et al. Amplification patterns of three genomic regions predict distant recurrence in breast carcinoma. *J Mol Diagn* **9**, 327-336 (2007).
9. Ring, B.Z. et al. Novel prognostic immunohistochemical biomarker panel for estrogen receptor-positive breast cancer. *Journal of clinical oncology : official journal of the American Society of Clinical Oncology* **24**, 3039-3047 (2006).
10. Whitehead, C.M., Nelson, R. & Hudson, P. Selection and optimization of a panel of early stage breast cancer prognostic molecular markers. *Mod Pathol* **17**, 50A (2004).
11. Desmedt, C. et al. Strong time dependence of the 76-gene prognostic signature for node-negative breast cancer patients in the TRANSBIG multicenter independent validation series. *Clin Cancer Res* **13**, 3207-3214 (2007).
12. Sotiriou, C. et al. Gene expression profiling in breast cancer: understanding the molecular basis of histologic grade to improve prognosis. *J Natl Cancer Inst* **98**, 262-272 (2006).
13. Liu, R. et al. The prognostic role of a gene signature from tumorigenic breast-cancer cells. *The New England journal of medicine* **356**, 217-226 (2007).
14. Ayers, M. et al. Gene expression profiles predict complete pathologic response to neoadjuvant paclitaxel and fluorouracil, doxorubicin, and cyclophosphamide chemotherapy in breast cancer. *Journal of clinical oncology : official journal of the American Society of Clinical Oncology* **22**, 2284-2293 (2004).
15. Nielsen, K.V. et al. The value of TOP2A gene copy number variation as a biomarker in breast cancer: Update of DBCG trial 89D. *Acta oncologica* **47**, 725-734 (2008).
16. Gu, F., Li, Z.H., Wang, C.Q., Yuan, Q.F. & Yan, Z.M. Effects of forkhead Box protein A1 on cell proliferation regulating and EMT of cervical carcinoma. *European review for medical and pharmacological sciences* **22**, 7189-7196 (2018).
17. Clark, J.W. et al. The potential role for prolactin-inducible protein (PIP) as a marker of human breast cancer micrometastasis. *Br J Cancer* **81**, 1002-1008 (1999).
18. Calaf, G.M., Echiburu-Chau, C., Zhao, Y.L. & Hei, T.K. BigH3 protein expression as a marker for breast cancer. *Int J Mol Med* **21**, 561-568 (2008).
19. Gonzalez, M.A. et al. Minichromosome maintenance protein 2 is a strong independent prognostic marker in breast cancer. *Journal of clinical oncology : official journal of the American Society of Clinical Oncology* **21**, 4306-4313 (2003).

20. Zhao, J. et al. Expression of Human Endogenous Retrovirus Type K Envelope Protein is a Novel Candidate Prognostic Marker for Human Breast Cancer. *Genes Cancer* **2**, 914-922 (2011).
21. Golmohammadi, R. & Pejhan, A. The prognostic value of the P53 protein and the Ki67 marker in breast cancer patients. *J Pak Med Assoc* **62**, 871-875 (2012).
22. Xu, L. et al. Expression status of cyclaseassociated protein 2 as a prognostic marker for human breast cancer. *Oncol Rep* **36**, 1981-1988 (2016).
23. Wu, K. et al. Expression of neuronal protein synuclein gamma gene as a novel marker for breast cancer prognosis. *Breast Cancer Res Treat* **101**, 259-267 (2007).
24. Levy, D. et al. Death-associated protein kinase loss of expression is a new marker for breast cancer prognosis. *Clin Cancer Res* **10**, 3124-3130 (2004).
25. McGovern, S.L., Qi, Y., Pusztai, L., Symmans, W.F. & Buchholz, T.A. Centromere protein-A, an essential centromere protein, is a prognostic marker for relapse in estrogen receptor-positive breast cancer. *Breast Cancer Res* **14**, R72 (2012).
26. Patel, A.N. et al. Mediator of DNA damage checkpoint protein 1 (MDC1) expression as a prognostic marker for nodal recurrence in early-stage breast cancer patients treated with breast-conserving surgery and radiation therapy. *Breast Cancer Res Treat* **126**, 601-607 (2011).
27. Frankel, L.B., Lykkesfeldt, A.E., Hansen, J.B. & Stenvang, J. Protein Kinase C alpha is a marker for antiestrogen resistance and is involved in the growth of tamoxifen resistant human breast cancer cells. *Breast Cancer Res Treat* **104**, 165-179 (2007).
28. Huang, J. et al. Y-box binding protein, YB-1, as a marker of tumor aggressiveness and response to adjuvant chemotherapy in breast cancer. *Int J Oncol* **26**, 607-613 (2005).
29. Kumar, R. et al. Serum 5-LOX: a progressive protein marker for breast cancer and new approach for therapeutic target. *Carcinogenesis* **37**, 912-917 (2016).
30. Nadler, Y. et al. Growth factor receptor-bound protein-7 (Grb7) as a prognostic marker and therapeutic target in breast cancer. *Ann Oncol* **21**, 466-473 (2010).
31. Gam, L.-H. Breast cancer and protein biomarkers. *World journal of experimental medicine* **2**, 86-91 (2012).
32. Yamashita, H. et al. Coexistence of HER2 over-expression and p53 protein accumulation is a strong prognostic molecular marker in breast cancer. *Breast Cancer Res* **6**, R24-30 (2004).
33. Youn, B., Kim, H.D. & Kim, J. Nm23-H1/nucleoside diphosphate kinase as a key molecule in breast tumor angiogenesis. *Expert opinion on therapeutic targets* **12**, 1419-1430 (2008).
34. Revillion, F. et al. Expression of the putative tumor suppressor gene PTPN13/PTPL1 is an independent prognostic marker for overall survival in breast cancer. *Int J Cancer* **124**, 638-643 (2009).
35. Dai, X., Liu, Z. & Zhang, S. Over-expression of EPS15 is a favorable prognostic factor in breast cancer. *Mol Biosyst* **11**, 2978-2985 (2015).
36. Dai, X. et al. WDR5 Expression Is Prognostic of Breast Cancer Outcome. *PLoS One* **10**, e0124964 (2015).
37. M, M.R. et al. Nectin-4: a new prognostic biomarker for efficient therapeutic targeting of primary and metastatic triple-negative breast cancer. *Ann Oncol* **28**, 769-776 (2017).
38. Dai, X. et al. Cooperation of DLC1 and CDK6 affects breast cancer clinical outcome. *G3 (Bethesda)* **5**, 81-91 (2014).
39. Dai, X., Fagerholm, R., Khan, S., Blomqvist, C. & Nevanlinna, H. INPP4B and RAD50 have an interactive effect on survival after breast cancer. *Breast Cancer Res Treat* **149**, 363-371 (2015).
40. Huang, L.L. et al. Potential role of miR-139-5p in cancer diagnosis, prognosis and therapy. *Oncol Lett* **14**, 1215-1222 (2017).
41. Shen, Y. et al. Prognostic and predictive values of long non-coding RNA LINC00472 in breast cancer. *Oncotarget* **6**, 8579-8592 (2015).

42. Xu, S.P. et al. The long non-coding RNA EPB41L4A-AS2 inhibits tumor proliferation and is associated with favorable prognoses in breast cancer and other solid tumors. *Oncotarget* **7**, 20704-20717 (2016).
43. Zhang, J.J., Guo, S.H. & Jia, B.Q. Down-regulation of long non-coding RNA MEG3 serves as an unfavorable risk factor for survival of patients with breast cancer. *European review for medical and pharmacological sciences* **20**, 5143-5147 (2016).
44. Zhang, X.F., Liu, T., Li, Y. & Li, S. Overexpression of long non-coding RNA CCAT1 is a novel biomarker of poor prognosis in patients with breast cancer. *Int J Clin Exp Patho* **8**, 9440-9445 (2015).
45. Iacoangeli, A. et al. BC200 RNA in invasive and preinvasive breast cancer. *Carcinogenesis* **25**, 2125-2133 (2004).
46. Yang, F. et al. A novel long non-coding RNA FGF14-AS2 is correlated with progression and prognosis in breast cancer. *Biochem Bioph Res Co* **470**, 479-483 (2016).
47. Basak, P. et al. Role of H19, a long non-coding RNA, in development of resistance to endocrine therapy in breast cancer cells. *Cancer Res* **77** (2017).
48. Cai, Y., He, J. & Zhang, D. Suppression of long non-coding RNA CCAT2 improves tamoxifen-resistant breast cancer cells' response to tamoxifen. *Mol Biol+* **50**, 725-730 (2016).
49. Pickard, M.R. & Williams, G.T. Regulation of apoptosis by long non-coding RNA GAS5 in breast cancer cells: implications for chemotherapy. *Breast Cancer Res Treat* **145**, 359-370 (2014).
50. Godinho, M.F. et al. BCAR4 induces antioestrogen resistance but sensitises breast cancer to lapatinib. *Br J Cancer* **107**, 947-955 (2012).
51. Shuai, P. et al. Long noncoding RNA MALAT1 can serve as a valuable biomarker for prognosis and lymph node metastasis in various cancers: a meta-analysis. *Springerplus* **5** (2016).
52. Xia, Y. et al. Targeting long non-coding RNA ASBEL with oligonucleotide antagonist for breast cancer therapy. *Biochem Biophys Res Commun* **489**, 386-392 (2017).
53. Liu, X.R. et al. Identification of high independent prognostic value of nanotechnology based circulating tumor cell enumeration in first-line chemotherapy for metastatic breast cancer patients. *Breast* **32**, 119-125 (2017).
54. Li, Y.H. et al. Inhibition of long non-coding RNA ROR reverses resistance to Tamoxifen by inducing autophagy in breast cancer. *Tumor Biol* **39** (2017).
55. Fang, X. et al. Twist2 contributes to breast cancer progression by promoting an epithelial-mesenchymal transition and cancer stem-like cell self-renewal. *Oncogene* **30**, 4707-4720 (2011).
56. Tanabe, Y. et al. Pathological features of triple-negative breast cancers that showed progressive disease during neoadjuvant chemotherapy. *Cancer science* **108**, 1520-1529 (2017).
57. Asmann, Y.W. et al. Detection of redundant fusion transcripts as biomarkers or disease-specific therapeutic targets in breast cancer. *Cancer Res* **72**, 1921-1928 (2012).
58. Boberg, D.R. et al. Copy number variation in ACHE/EPHB4 (7q22) and in BCHE/MME (3q26) genes in sporadic breast cancer. *Chemico-biological interactions* **203**, 344-347 (2013).
59. Moscovich, M. et al. Dystonia, facial dysmorphism, intellectual disability and breast cancer associated with a chromosome 13q34 duplication and overexpression of TFDP1: case report. *BMC Med Genet* **14**, 70 (2013).
60. Wu, D. et al. Intron 3 sixteen base pairs duplication polymorphism of p53 contributes to breast cancer susceptibility: evidence from meta-analysis. *PLoS One* **8**, e61662 (2013).
61. Huang, K.T. et al. Assessment of DNA methylation profiling and copy number variation as indications of clonal relationship in ipsilateral and contralateral breast cancers to distinguish recurrent breast cancer from a second primary tumour. *BMC cancer* **15**, 669 (2015).
62. Evron, E. et al. Detection of breast cancer cells in ductal lavage fluid by methylation-specific PCR. *Lancet* **357**, 1335-1336 (2001).
63. Widschwendter, M. et al. Association of breast cancer DNA methylation profiles with hormone

receptor status and response to tamoxifen. *Cancer Res* **64**, 3807-3813 (2004).
64. Zhou, Y. et al. Diagnostic Accuracy of PIK3CA Mutation Detection by Circulating Free DNA in Breast Cancer: A Meta-Analysis of Diagnostic Test Accuracy. *PLoS One* **11**, e0158143 (2016).
65. Wooster, R. et al. Identification of the breast cancer susceptibility gene BRCA2. *Nature* **378**, 789-792 (1995).
66. Lebok, P. et al. Partial PTEN deletion is linked to poor prognosis in breast cancer. *BMC cancer* **15**, 963 (2015).
67. Han, M.-R. et al. Evaluation of genetic variants in high and moderate-penetrance breast cancer susceptibility genes in East Asians. *Cancer Res* **75**, doi:10.1158/1538-7445.AM2015-2780 (2015).
68. Han MR et al. Evaluating genetic variants associated with breast cancer risk in high and moderate-penetrance genes in Asians. *Carcinogenesis* **38**, 511-518 (2017).
69. Samia Shabnaz et al. Breast cancer risk in relation to TP53 codon 72 and CDH1 gene polymorphisms in the Bangladeshi women. *Tumor Biol* **37**, 7229-7237 (2016).
70. Uzunoglu, H. et al. Association of the nibrin gene (NBN) variants with breast cancer. *Biomedical Reports* **4**, 369-373 (2016).
71. Johnson, N. et al. Counting potentially functional variants in BRCA1, BRCA2 and ATM predicts breast cancer susceptibility. *Human Molecular Genetics* **16**, 1051-1057 (2007).
72. Peterlongo, P. et al. FANCM c.5791C>T nonsense mutation (rs144567652) induces exon skipping, affects DNA repair activity and is a familial breast cancer risk factor. *Hum Mol Genet* **24**, 5345-5355 (2015).
73. Harlid, S. et al. Combined effect of low-penetrant SNPs on breast cancer risk. *Br J Cancer* **106**, 389-396 (2012).
74. Frank, B. et al. BRIP1 (BACH1) variants and familial breast cancer risk: a case-control study. *BMC cancer* **7** (2007).
75. Charoenchokthavee, W., Ayudhya, D.P., Sriuranpong, V. & Areepium, N. Effects of SULT1A1 Copy Number Variation on Estrogen Concentration and Tamoxifen-Associated Adverse Drug Reactions in Premenopausal Thai Breast Cancer Patients: A Preliminary Study. *Asian Pacific journal of cancer prevention : APJCP* **17**, 1851-1855 (2016).
76. Kuiper, R.P., Ligtenberg, M.J., Hoogerbrugge, N. & Geurts van Kessel, A. Germline copy number variation and cancer risk. *Curr Opin Genet Dev* **20**, 282-289 (2010).
77. Kapahi, R. et al. Association of -2549 insertion/deletion polymorphism of vascular endothelial growth factor with breast cancer in North Indian patients. *Genet Test Mol Biomarkers* **17**, 242-248 (2013).
78. Hashemi, M. et al. A 40-bp insertion/deletion polymorphism of Murine Double Minute2 (MDM2) increased the risk of breast cancer in Zahedan, Southeast Iran. *Iran Biomed J* **18**, 245-249 (2014).
79. Haghi, M., Hosseinpour Feizi, M.A., Sadeghizadeh, M. & Lotfi, A.S. 14-bp Insertion/Deletion Polymorphism of the HLA-G gene in Breast Cancer among Women from North Western Iran. *Asian Pacific journal of cancer prevention : APJCP* **16**, 6155-6158 (2015).
80. Huang, X., Yang, Y., Cui, Z.W., Wang, J. & Gao, L.B. A functional insertion/deletion polymorphism in the IL1A gene is associated with decreased risk of breast cancer. *Genet Mol Res* **15** (2016).
81. Hashemi, M. et al. Association between Programmed Cell Death 6 Interacting Protein Insertion/Deletion Polymorphism and the Risk of Breast Cancer in a Sample of Iranian Population. *Dis Markers* **2015**, 854621 (2015).
82. Jakubowska, A. et al. Breast cancer risk reduction associated with the RAD51 polymorphism among carriers of the BRCA1 5382insC mutation in Poland. *Cancer Epidemiol Biomarkers Prev* **12**, 457-459 (2003).
83. Han, Y. et al. APOBEC3 deletion increases the risk of breast cancer: a meta-analysis. *Oncotarget* **7**, 74979-74986 (2016).

84. Stroun, M. et al. Neoplastic characteristics of the DNA found in the plasma of cancer patients. *Oncology* **46**, 318-322 (1989).
